# The aryl hydrocarbon receptor pathway controls matrix metalloproteinase-1 and collagen levels in human orbital fibroblasts

**DOI:** 10.1038/s41598-020-65414-1

**Published:** 2020-05-21

**Authors:** Elisa Roztocil, Christine L. Hammond, Mithra O. Gonzalez, Steven E. Feldon, Collynn F. Woeller

**Affiliations:** 1 Flaum Eye Institute, University of Rochester, Rochester, New York 14642 USA; 2 Department of Environmental Medicine School of Medicine and Dentistry, University of Rochester, Rochester, New York 14642 USA

**Keywords:** Proteases, Proteins, Transcription, Biochemistry, Molecular biology, Cell biology, Mechanisms of disease, Proteolysis, Drug discovery, Target identification, Target validation, Eye diseases, Immunological disorders

## Abstract

Thyroid eye disease (TED) affects 25–50% of patients with Graves’ Disease. In TED, collagen accumulation leads to an expansion of the extracellular matrix (ECM) which causes destructive tissue remodeling. The purpose of this study was to investigate the therapeutic potential of activating the aryl hydrocarbon receptor (AHR) to limit ECM accumulation *in vitro*. The ability of AHR to control expression of matrix metalloproteinase-1 (MMP1) was analyzed. MMP1 degrades collagen to prevent excessive ECM. Human orbital fibroblasts (OFs) were treated with the pro-scarring cytokine, transforming growth factor beta (TGFβ) to induce collagen production. The AHR ligand, 6-formylindolo[3,2b]carbazole (FICZ) was used to activate the AHR pathway in OFs. MMP1 protein and mRNA levels were analyzed by immunosorbent assay, Western blotting and quantitative PCR. MMP1 activity was detected using collagen zymography. AHR and its transcriptional binding partner, ARNT were depleted using siRNA to determine their role in activating expression of MMP1. FICZ induced MMP1 mRNA, protein expression and activity. MMP1 expression led to a reduction in collagen 1A1 levels. Furthermore, FICZ-induced MMP1 expression required both AHR and ARNT, demonstrating that the AHR-ARNT transcriptional complex is necessary for expression of MMP1 in OFs. These data show that activation of the AHR by FICZ increases MMP1 expression while leading to a decrease in collagen levels. Taken together, these studies suggest that AHR activation could be a promising target to block excessive collagen accumulation and destructive tissue remodeling that occurs in fibrotic diseases such as TED.

## Introduction

Thyroid eye disease (TED) affects 25–50% of patients with Graves’ disease, which is characterized by the presence of activating autoantibodies to the thyroid stimulating hormone receptor causing hyperthyroidism^1^. TED results from excessive inflammation and remodeling of the orbital soft tissue. Tissue expansion leads to exophthalmos (bulging of the eyes) and eyelid retraction that can cause irritation, pain and vision impairment. Inflammatory cells including T and B lymphocytes, mast cells and macrophages infiltrate the orbit and activate orbital fibroblasts (OFs) to form lipid accumulating adipocytes and/or scar-forming myofibroblasts^2^. Activated OFs and myofibroblasts produce high levels of hyaluronan and collagen that accumulate in the extracellular matrix (ECM) to promote destructive tissue enlargement and reorganization^3^. The newly deposited ECM also contributes to greater recruitment and retention of inflammatory cells that promote sustained OF and myofibroblast activation, propelling a feed-forward cycle of disease. Unfortunately, few therapies effectively block TED tissue remodeling. Standard treatment options in TED include use of corticosteroids to limit inflammation (which is only effective in some cases) and beyond that surgical measures are used to eliminate excessive soft orbital tissue^4^. Therefore, new, targeted therapies are needed to limit or reverse destructive tissue scar formation and remodeling observed in TED.

Matrix metalloproteinases (MMPs) are a family of zinc-dependent proteases that degrade collagen and other ECM proteins to regulate tissue structure and remodeling. Currently, there are 25 known MMPs that localize on the surface of cells or reside in the extracellular milieu^5^. The first MMP family member identified was MMP1, which is also called interstitial collagenase, and resides in the extracellular space. MMP1 hydrolyzes collagen fibers (types I and III) present in connective tissue^6^. Collagen I (COL1A1), is highly produced by activated OFs and is a major component of extracellular scar tissue^7,8^. Other MMPs that degrade collagen include MMP2 and MMP9. MMP2 and 9 target collagen types I and IV and IV and V, respectively. Given their different substrate preferences and expression patterns, MMPs have distinct roles in the regulation of collagen content in the ECM during growth, development, injury response and disease progression^6^. Transforming growth factor beta (TGFβ), a master regulator of myofibroblast differentiation, wound healing and tissue scarring, regulates expression of some MMPs. Both MMP2 and MMP9 are increased by TGFβ in epithelial cells and cardiac fibroblasts^9,10^. Furthermore, MMP2 and MMP9 have a pro-fibrotic role in kidney and cardiac scar formation^11,12^. In contrast, MMP1 expression is reduced by TGFβ in dermal fibroblasts^13^. Some studies suggest that increasing MMP1 expression or delivery of MMP1 protein directly to scar tissue may be a therapeutic opportunity to promote resolution and/or clearance of excessive collagen I fibers and scar tissue^14,15^.

The aryl hydrocarbon receptor (AHR) is a ligand activated transcription factor that binds a diverse range of synthetic and naturally derived aromatic hydrocarbons. Upon ligand binding, the AHR is imported into the nucleus where it interacts with the AHR nuclear translocator protein 1 (ARNT) to activate transcription of target genes^16^. AHR-dependent genes include several cytochrome P450 enzymes, such as CYP1B1 and CYP1A1, which metabolize and clear xenobiotic polycyclic aromatic hydrocarbons (PAHs). AHR also induces expression of its own repressor protein, aryl hydrocarbon receptor repressor (AHRR) as a form of negative feedback on the pathway. While AHR is a ligand activated transcription factor that regulates gene transcription in the nucleus, it also carries out several different functions in the cytoplasm. For example, it can regulate Ca2+ flux and Src activity in certain cell types to control inflammatory signaling^17,18^. Thus, the AHR plays important roles in both cytoplasmic and nuclear compartments.

Studies using *Ahr* knockout animals revealed an important role for the receptor in regulating inflammatory and immune responses^19^. Work by Puga and colleagues have revealed a key regulatory link by which the AHR regulates numerous signaling pathways, including TGFβ-dependent signaling^20^. *Ahr* knockout mice display an increased wound healing response that involves promoting keratinocyte migration and acceleration of skin re-epithelialization after injury^21^. The AHR also reduces ECM accumulation in retinal pigment epithelial cells (RPE) and *Ahr* knockout animals display increased matrix accumulation and age-related macular degeneration compared to wild-type controls^22^. We reported that the naturally occurring tryptophan derivative, 6-formylindolo[3,2b]carbozole (FICZ) mitigates TED myofibroblast formation and TGFβ-dependent cell proliferation^23^. Furthermore, the widely used proton pump inhibitors (PPIs) esomeprazole and lansoprazole activate the AHR and reduce OF proliferation and TGFβ-dependent myofibroblast formation^24^.

In the current study, the role of AHR activation in regulating expression of MMP1, 2 and 9 was investigated. We determined that FICZ induces MMP1 mRNA and protein expression in human OFs. Furthermore, we found that MMP1 expression requires both AHR and ARNT, revealing that the AHR-ARNT transcriptional complex is necessary for production of MMP1 in OFs. AHR induced MMP1 expression leads to a reduction in collagen 1 accumulation suggesting that AHR activation may be a promising target to block the unwanted ECM production and tissue remodeling observed in TED.

## Results

### TGFβ reduces MMP1, whereas FICZ increases MMP1 levels in GOFs

**Figure 1:**
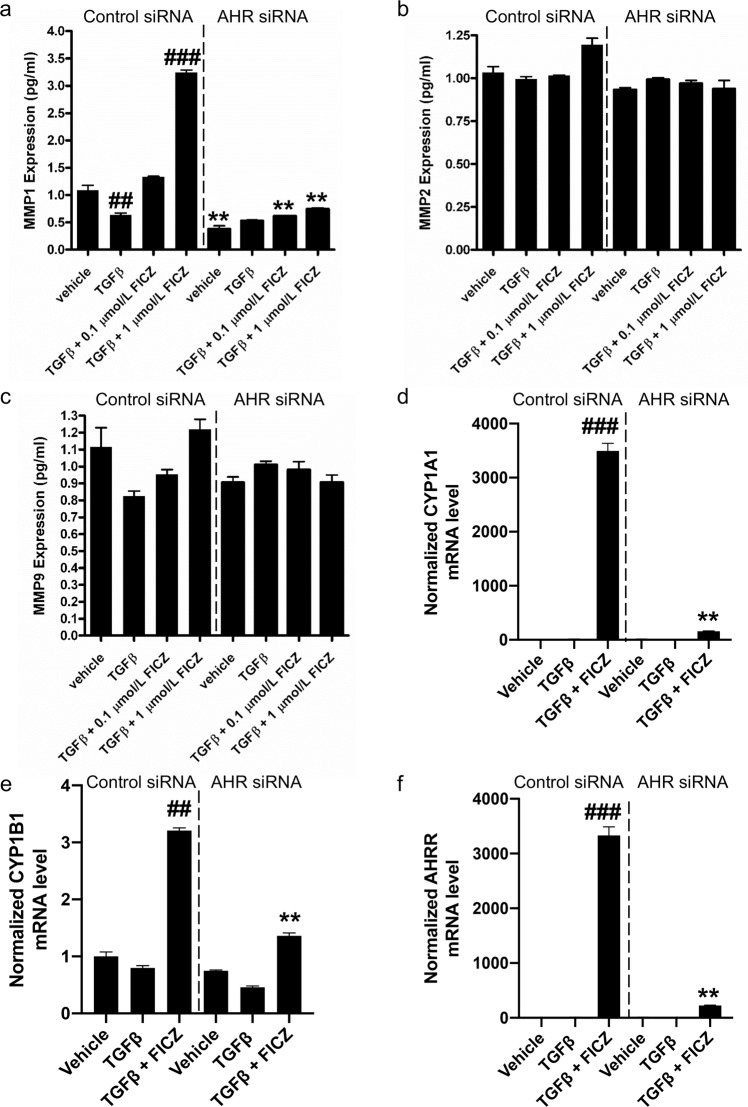
MMP1 levels, but not MMP2 or MMP9 levels are regulated by the AHR in Graves’ orbital fibroblasts (GOFs). GOFs were treated with non-specific siRNA (control) or *AHR* siRNA for 48 hours. Afterwards, cells were incubated with 0.1% FBS DMEM medium containing either vehicle (DMSO), TGFβ, TGFβ + 0.1 μM FICZ or TGFβ + 1 μM FICZ for 24 hours. Cell culture supernatants were then collected and analyzed by fluorescent based immunoassay (Luminex) for MMP1 **(a)**, MMP2 **(b)** or MMP9 **(c)**. MMP1 levels were reduced by TGFβ and increased by FICZ. *AHR* siRNA attenuated MMP1 induction by FICZ. MMP2 and MMP9 levels were not significantly altered by any treatments or by *AHR* siRNA. To confirm that FICZ activated AHR dependent gene expression and AHR siRNA successfully blocked AHR dependent gene expression, canonical AHR dependent genes were analyzed by qPCR in samples treated with vehicle, TGFβ or TGFβ + 1 μM FICZ for 24 hours. Total RNA was isolated and analyzed by RT-qPCR. Normalized levels of CYP1A1 mRNA (**d**), CYP1B1 mRNA (**e**) and AHRR mRNA (**f**) are shown. FICZ significantly induced expression of all three canonical AHR dependent genes in GOFs while AHR siRNA dramatically attenuated the effect of FICZ on gene expression. The experiment was performed in 3 different strains of GOFs. ^##^p < 0.01, ^###^p < 0.001 versus vehicle treatment. **p < 0.01 in AHR vs control siRNA samples with the same treatment.

Matrix metalloproteinases (MMPs) are extracellular proteinases that catalyze the degradation and turnover of target proteins to regulate the architecture of the ECM. In TED, fibrous collagen accumulation contributes to excessive ECM causing tissue reorganization, inflammatory cell retention and ultimately, tissue damage^25^. Previous work revealed that AHR ligands block TGFβ-induced myofibroblast formation in GOFs^23^. To determine if AHR can regulate MMPs that control collagen accumulation, MMP1, 2 and 9 levels were analyzed in GOF conditioned culture medium (Fig. 1). GOFs were treated with the AHR ligand FICZ (0.1 μM or 1 μM) +/− TGFβ (5 ng/mL) for 24 hours and then culture supernatants were collected for analysis. The experiment was performed in cells treated with either control siRNA or *AHR* siRNA (to deplete cells of AHR) to determine if changes in MMPs required AHR. In control siRNA treated GOFs, MMP1 levels were significantly reduced by TGFβ (Fig. 1a). Low dose FICZ (0.1 μM) treatment attenuated the ability of TGFβ to reduce MMP1 levels and 1 μM FICZ significantly induced MMP1 levels by over 3-fold compared to vehicle treatment. Depletion of AHR prevented MMP1 production in vehicle and FICZ treated GOFs. While MMP1 levels in GOFs were increased by FICZ in an AHR dependent manner, MMP2 and MMP9 levels were not significantly changed by FICZ, TGFβ or AHR expression (Fig. 1b,c). To confirm that FICZ (1 μM) activated AHR dependent gene expression in TGFβ-treated samples, canonical AHR dependent genes were analyzed by qPCR. Normalized levels of *CYP1A1*, *CYP1B1*, *AHRR* mRNA are shown (Fig. 1d–f). FICZ significantly induced expression of all three AHR-dependent genes in GOFs while *AHR* siRNA dramatically attenuated the effect of FICZ on gene expression.

**Figure 2:**
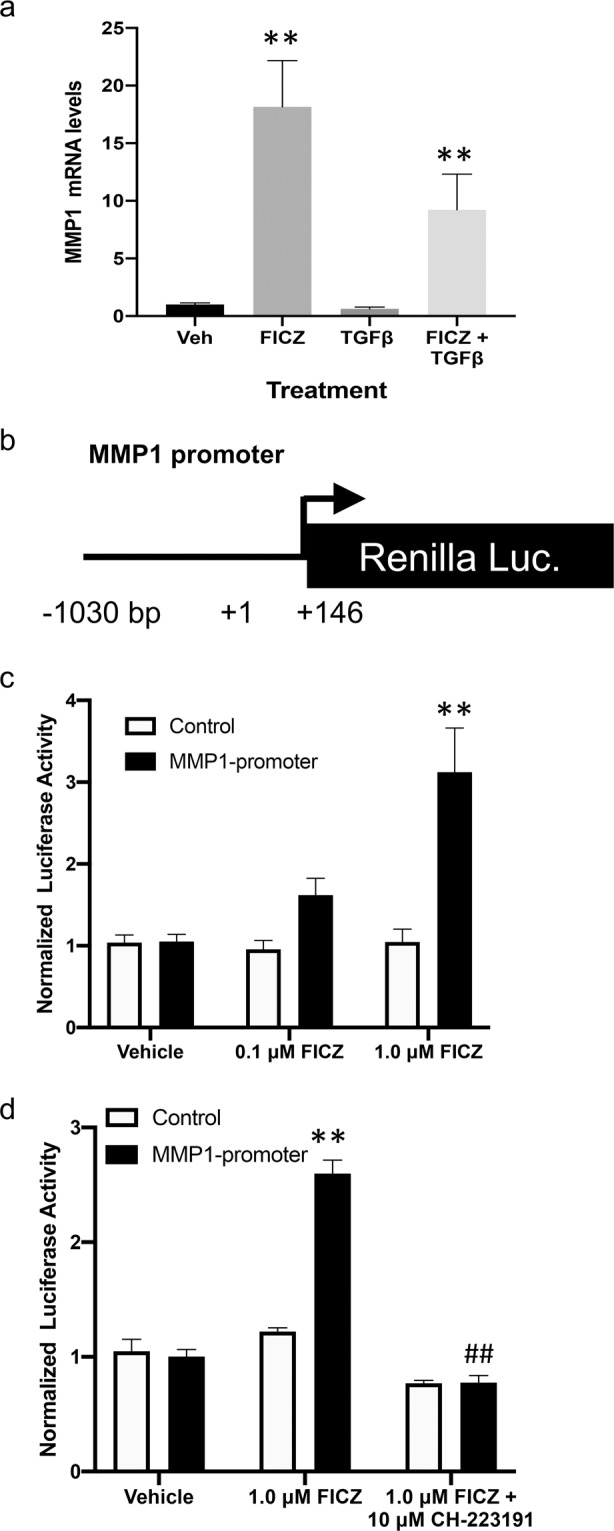
MMP1 expression is induced by the AHR ligand FICZ in GOFs. (**a**) Three different GOF strains were grown under normal culture conditions then serum starved for 48 hours. Cells were then pre-treated with 1 μM FICZ for 1–2 hours followed by the addition of 5 ng/ml TGFβ to promote myofibroblast differentiation. After 24 hours, cells were collected and RNA was isolated for qPCR analysis of MMP1 mRNA and 18 S rRNA (control) levels. **(b)** Diagram of the *MMP1* promoter reporter construct. The human *MMP1* promoter region (−1030 to +146) is inserted upstream of the Renilla luciferase open reading frame. **(c)** Reporter constructs with the *MMP1* promoter or without the promoter were introduced into three different GOFs strains and then treated with DMSO (vehicle), or FICZ (0.1 or 1.0 μM) for 16 hours after transfection. Then cells were lysed and Renilla activity was measured. (**d**) To determine if the ability of FICZ to induce MMP1-promoter activity is dependent upon AHR, the potent AHR inhibitor, CH-233191 (10 μM) was added 30 minutes prior to FICZ (1 μM). After 16 hours, cells were lysed and Renilla activity measured. Renilla activity levels were normalized to total protein and vehicle samples for both MMP1 and control promoters were set to 1.0. **p < 0.01 versus vehicle treatment, ^##^p < 0.01 vs FICZ alone treatment.

Next, the ability of FICZ to induce *MMP1* mRNA levels was tested. GOFs were cultured in the presence or absence of 1 μM FICZ +/− TGFβ for 24 hours before cell extracts were harvested and analyzed by qPCR for *MMP1* mRNA levels. FICZ treatment led to a ~17-fold induction of *MMP1* mRNA levels (Fig. 2a). TGFβ treatment decreased baseline expression of *MMP1* mRNA to ~60% of vehicle treated cells (similar to the decrease observed in MMP1 protein levels from conditioned culture medium). TGFβ also reduced the ability of FICZ to stimulate *MMP1* mRNA by around 40%. Nevertheless, *MMP1* mRNA was induced ~10-fold in TGFβ-FICZ treated cells compared to vehicle treatment.

To determine if FICZ and AHR directly activate *MMP1* mRNA transcription, a Renilla luciferase reporter plasmid containing the proximal human *MMP1* promoter region (−1030 to +146 bp of the human *MMP1* locus) was obtained (Fig. 2b). The *MMP1* promoter plasmid construct or a control Renilla construct without the *MMP1* promoter were introduced into GOFs by transient transfection. After transfection, cells were treated with vehicle, 0.1 μM or 1 μM FICZ for 16 hours. Cells were harvested and Renilla Luciferase activity was measured and normalized to vehicle treated cells (Fig. 2c). FICZ treatment led to a dose-dependent increase in *MMP1* promoter activity whereas FICZ had no effect on the control construct activity. To determine if the ability of FICZ to induce *MMP1*-promoter activity is dependent upon AHR ligand binding, the specific and potent small molecule AHR inhibitor, CH-223191 was used in the reporter assay (Fig. 2d)^26^. CH-223191 (10 μM) completely blocked the ability of FICZ to induce the *MMP1* promoter construct while the control promoter construct was not affected by the inhibitor. This data suggests that FICZ induces *MMP1* promoter activity dependent upon the AHR.

### MMP1 levels and activity are induced by FICZ in orbital fibroblasts

**Figure 3:**
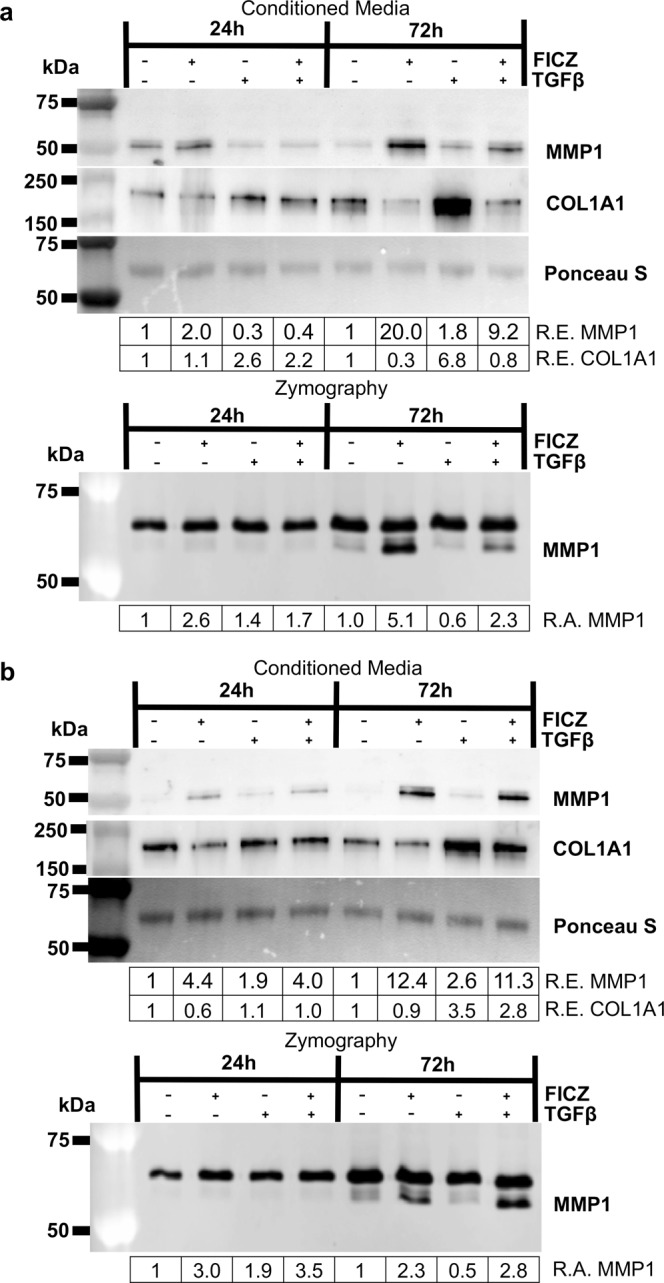
The AHR ligand FICZ increases MMP1 production and activity and decreases collagen 1 levels. GOFs were treated with the AHR ligand FICZ, 1 μM, for 1–2 hours before additional treatment with or without 5 ng/ml TGFβ to promote myofibroblast differentiation. At both 24 and 72-hour timepoints, media was collected and analyzed by Western blotting and Collagen I zymography. **(a)** Upper panel, equal amounts of protein from culture supernatant were loaded. Ponceau S staining, which shows a predominant band around 50 kDa, was used to confirm equal loading. MMP1 and collagen 1 (COL1A1) protein levels were determined by using specific antibodies as stated in the Methods section. Relative protein expression (R.E.) based on densitometry is shown below each blot. FICZ increases MMP1 levels while simultaneously decreasing COL1A1 levels particularly after 72 hours. Lower panel, the same culture supernatants were analyzed by zymography with collagen I as substrate. Relative MMP1 activity (R.A.) based on densitometry of the inverted zymography gels is shown below each image. Importantly, only the lower band represents MMP1 activity. **(b)** An additional GOF strain showing similar changes in MMP1 and collagen levels (upper panel) and MMP1 activity (lower panel). These experiments were performed in six different GOF strains and three different NOF strains with similar responses seen in all strains. Additional strain data is presented in Supplemental Figs. 1–3. Full length blots for Fig. (3a,b) can be seen in Supplemental Figs. 14 and 15, with full length blots from additional the strains in Supplemental Figs. 21–23.

Additional studies were performed in both GOFs and NOFs to further confirm that FICZ can induce MMP1 expression and activity. Fibroblasts were treated with vehicle or 1 μM FICZ +/− TGFβ for 24 or 72 hours. Conditioned media were collected and analyzed by Western blotting for MMP1 and collagen 1A1 (COL1A1) levels. MMP1 activity was analyzed by collagen I zymography. Two different GOF strains are shown in Fig. 3a and in Fig. 3b (additional GOF and NOF strains are shown in Supp Figs. 1–3). In all OF strains tested, MMP1 levels and activity were increased by the addition of FICZ, with the 72-hour timepoint showing a larger induction compared to the 24-hour timepoint (Fig. 3a,b, upper panels). At 72 hours, COL1A1 levels in the supernatant were increased by TGFβ. COL1A1 levels in both vehicle and TGFβ treated cells were attenuated by FICZ treatment.

**Figure 4:**
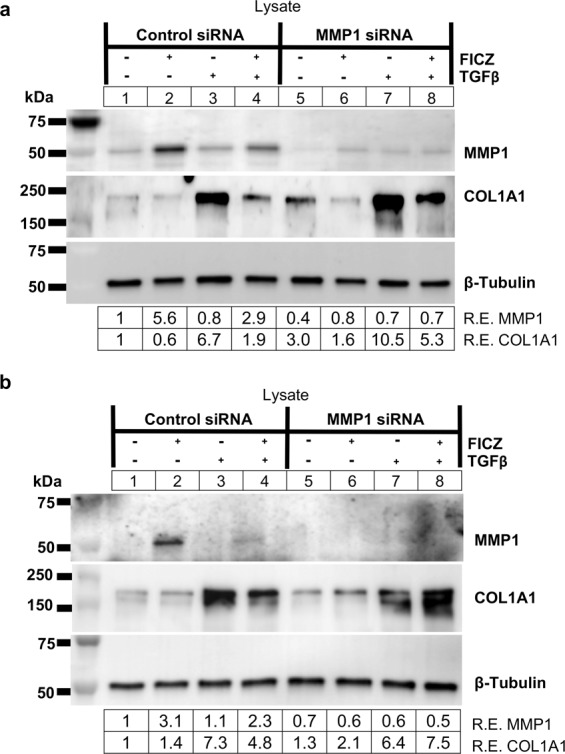
MMP1 knockdown increases collagen I levels in GOFs. (**a**) GOFs were treated with control or *MMP1* specific siRNA for 48 hours and then treated with either vehicle (DMSO) or the AHR ligand FICZ (1 µM) for 1 hour before addition of TGFβ (5 ng/mL) as indicated. (upper panel) After 72 hours of TGFβ treatment, cell extracts were isolated and analyzed by Western blot for MMP1, Collagen 1 (COL1A1) and β-tubulin (loading control). Relative protein expression (R.E.) based on densitometry are listed below the images. *MMP1* siRNA reduced MMP1 protein expression and increased COL1A1 compared to control siRNA levels. **(b)** shows an additional strain with increased COL1A1 expression in the presence of *MMP1* siRNA. Full length blots for **(a,b)** can be found in Supplemental Fig. 16. An additional strain was used for this experiment in Supplemental Fig. 4 with its corresponding full-length blots in Supplemental Fig. 24.

To further test if MMP1 expression leads to a reduction in COL1A1 levels, *MMP1* siRNA was used to deplete MMP1 in GOFs. Three different GOF strains were treated with control or *MMP1* specific siRNA for 48 hours before treatment with vehicle or 1 μM FICZ +/− TGFβ for an additional 72 hours (Fig. 4a,b and Supp Fig. 4). Cell lysates were collected and analyzed by Western blotting for MMP1, COL1A1 and tubulin (control). In control siRNA treated samples, FICZ increased MMP1 expression while TGFβ increased COL1A1 levels. Additionally, in control siRNA treatment, FICZ attenuated the ability of TGFβ to increase COL1A1. In *MMP1* siRNA treated samples, MMP1 levels were reduced by 60% in vehicle treated samples and ~2 to 3-fold in FICZ treated samples compared to control siRNA (Fig. 4a,b). Interestingly, in *MMP1* siRNA treated samples, COL1A1 levels were elevated compared to their corresponding control siRNA samples. Furthermore, the ability of FICZ to reduce COL1A1 levels was impaired in *MMP1* siRNA samples compared to control siRNA. These results show that MMP1 expression blocks COL1A1 accumulation in GOFs.

**Figure 5:**
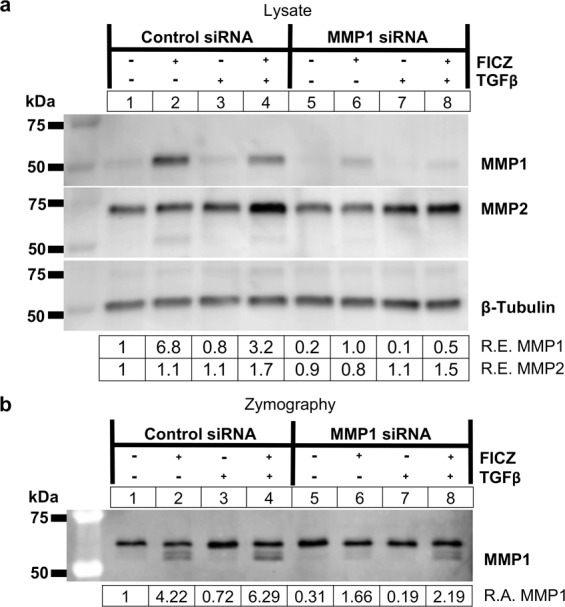
MMP1 knockdown attenuates FICZ mediated MMP1 production and activity. (**a**) GOFs were treated with control or *MMP1* specific siRNA for 48 hours and then treated with either vehicle (DMSO) or the AHR ligand FICZ (1 µM), and TGFβ (5 ng/mL) as indicated. (upper panel) After 72 hours of TGFβ treatment, cell extracts were isolated and analyzed by Western blot for MMP1, MMP2, and β-tubulin (loading control). Relative protein expression (R.E.) based on densitometry are listed below the images. *MMP1* siRNA reduced MMP1 protein expression to less than 15% of control siRNA levels. (**b**) Corresponding supernatants were also collected and analyzed using collagen I zymography. Relative MMP1 activity (R.A.) based on densitometry of the inverted zymography gel is shown below the image. The lower band, which represents MMP1 activity, is reduced more than 2-fold by *MMP1* siRNA. The top band, which corresponds to the molecular weight of MMP2, is not altered by *MMP1* siRNA. The experiment was also performed in additional GOF and NOF strains for comparison and these are shown in Supplemental Figs. 5 and 6. Full length blots for **a,b** are included in Supplemental Fig. 17. Full length blots for Supplemental Figs. 5 and 6 can be seen in Supplemental Figs. 25 and 26.

While our results show that MMP1 is able to reduce COL1A1 levels in GOFs, collagen I can be degraded by both MMP1 and MMP2^27^, and collagen I zymography can detect activity of both of these MMPs *in vitro*. Results presented in Fig. 1 show that MMP1 and MMP2 are both expressed in orbital fibroblasts. MMP2 appears to be constitutively expressed while MMP1 expression is regulated by TGFβ, AHR and FICZ. Additionally, since the collagen I zymography results revealed an additional band that is constitutively present above the inducible MMP1 signal (Fig. 3), *MMP1* siRNA was used to test specificity of both Western blotting and zymography assays (Fig. 5). GOFs were treated with control or *MMP1* specific siRNA for 48 hours before treatment with vehicle or 1 μM FICZ +/− TGFβ for an additional 72 hours. Afterwards, cell lysates were harvested for Western blotting (Fig. 5a) and cell culture supernatants were analyzed by collagen I zymography (Fig. 5b). Western blotting shows that *MMP1* siRNA reduced both basal and FICZ induced MMP1 expression by ~80% compared to control siRNA cells, regardless of treatment condition. MMP2 expression was largely unaltered by treatments or *MMP1* siRNA. Additionally, culture supernatants analyzed by zymography show that *MMP1* siRNA reduces MMP1 activity by 60% or more compared to control siRNA in all treatment conditions. The upper band in zymography assays is not reduced by *MMP1* siRNA, however, suggesting this band corresponds to a different MMP, most likely MMP2. Similar results were observed in additional OF strains (Supp Figs. 5 and 6). Furthermore, recombinant MMP1 and MMP2 proteins were tested in collagen I zymography assays and both proteinases showed collagenase activity (Supp Fig. 7a). These results suggest the upper band observed in the zymography gels is constitutively expressed MMP2, and that MMP1 is specifically induced by FICZ. As additional evidence showing the upper band in these zymography assays is MMP2, *MMP2* cDNA (or a control plasmid) were introduced into HEK293FT cells (Supp Fig. 7b). Culture supernatant from these cells showed increased zymography activity at the size of MMP2 and not at MMP1(Supp Fig. 7c) in HEK293FT cells that express exogenous MMP2. Even though MMP2 can degrade collagen I in zymography assays, it does not appear to degrade COL1A1 levels in orbital fibroblasts as demonstrated by the accumulation of COL1A1 in TGFβ treated orbital fibroblasts from Figs. 3 and 4.

### AHR and ARNT are required for FICZ induced MMP1 expression and activity

**Figure 6:**
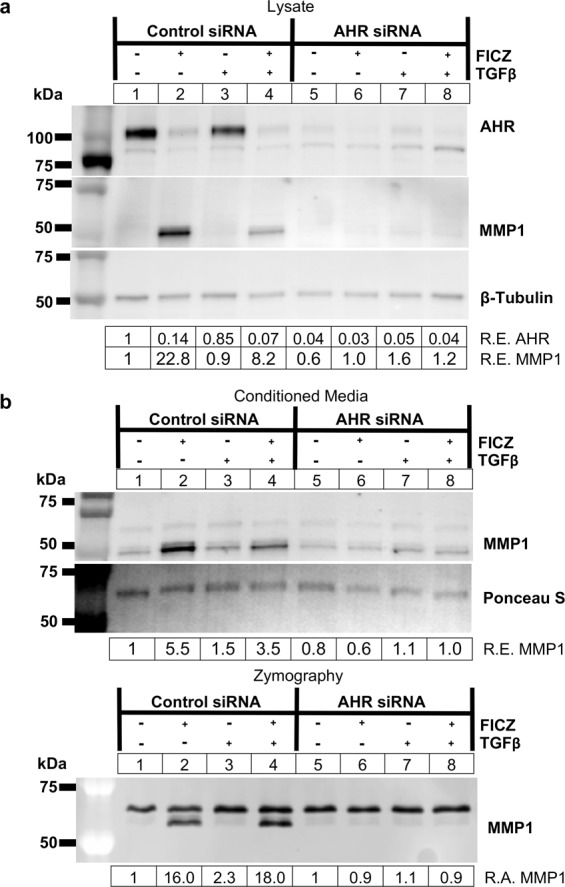
FICZ-induced MMP1 expression and activity occurs in an AHR-dependent manner. GOFs were treated with control or *AHR* specific siRNA for 48 hours and then treated with either vehicle (DMSO) or the AHR ligand FICZ (1 µM), and TGFβ (5 ng/mL) as indicated. **(a)** After 72 hours of TGFβ treatment, cell extracts were isolated and analyzed by Western blot for AHR, MMP1, and β-tubulin (loading control). Relative protein expression (R.E.) based on densitometry are listed below the images. *AHR* siRNA reduced AHR protein expression to less than 5% of control siRNA levels for all treatments tested. **(b)** Corresponding supernatants were also collected and analyzed using Western blotting (upper panel) and collagen I zymography (lower panel). Relative MMP1 levels (R.E.) based on densitometry are shown below the blot images. In the bottom panel, relative MMP1 activity (R.A.) based on densitometry of the inverted zymography gel is shown. The lower band, which represents MMP1 activity, is reduced in FICZ treated samples by more than 10-fold by *AHR* siRNA. The top band, which corresponds to the molecular weight of MMP2, is not altered by *AHR* siRNA. The experiment was also performed in additional GOF and NOF strains for comparison and data are shown in Supplemental Figs. 8–12. Full length blots for Fig. 6 can be seen in Supplemental Fig. 18 while full length blots for the additional strains are included in Supplemental Figs. 27–31.

Our data show that MMP1 production requires AHR expression, however, AHR can function both in the cytoplasm and in the nucleus through different mechanisms. To test if the mechanism whereby FICZ induces MMP1 in orbital fibroblasts requires AHR transcriptional activity, the AHR and ARNT transcription factors were depleted using gene specific siRNA. First, GOFs were plated and treated with control or *AHR* specific siRNA for 48 hours before treatment with vehicle or 1 μM FICZ +/− TGFβ for an additional 72 hours. Cell lysates and culture medium were collected and analyzed by Western blotting and collagen zymography (Fig. 6). *AHR* siRNA resulted in a greater than 95% depletion of AHR protein levels compared to control siRNA (Fig. 6a). Similar to the immunosorbent assays on culture medium and qPCR results on cell lysates from Fig. 1, AHR depletion by *AHR* siRNA blocked the ability of FICZ to induce MMP1 expression. Analysis of the culture medium revealed that MMP1 levels were not increased by FICZ in *AHR* siRNA treated cells whereas in control siRNA treated cells, MMP1 levels were induced 3.5 to 5.5-fold over vehicle (Fig. 6b, upper panel). Likewise, MMP1 activity was not induced by FICZ in *AHR* siRNA treated cells whereas in control siRNA treated cells, FICZ induced MMP1 by 16 to18-fold as detected in collagen I zymography (Fig. 6b, lower panel). These data further confirm the results presented in Fig. 1. Additional data from 4 GOF strains and 1 NOF strain show similar results (Supp Figs. 8–12).

**Figure 7:**
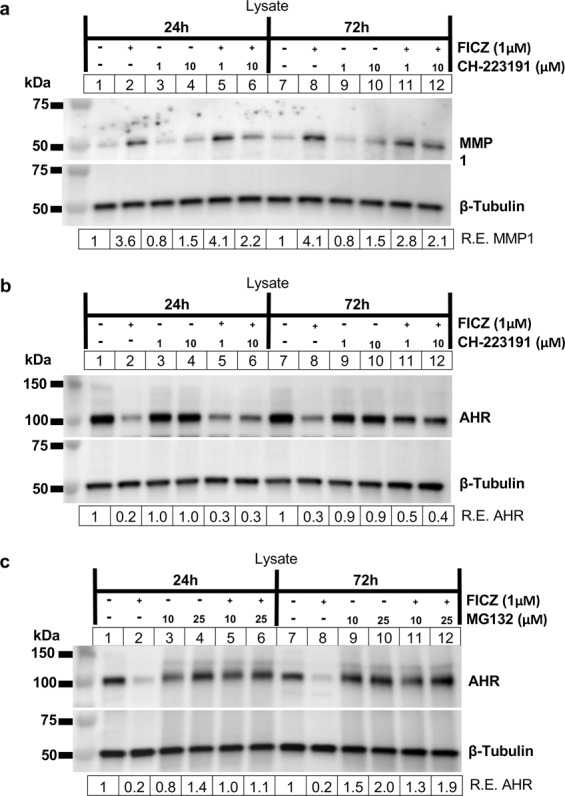
FICZ-induced MMP1 expression is blocked by CH-223191 and AHR ligand-induced AHR protein degradation is blocked by CH-223191 and proteasome inhibitor MG132. (**a**) GOFs were plated and treated with vehicle or CH-223191 (at 1 or 10 μM) for 1 hour before treatment with vehicle or 1 μM FICZ for an additional 24 or 72 hours. CH-223191 was added every 24 hours in the 72-hour samples. Cell lysates were collected and analyzed by Western blotting for MMP1 and β-tubulin (loading control) **(b)**. Cells treated as in **(a)** were analyzed for AHR and β-tubulin (loading control) by Western blot. **(c)** GOFs were plated and treated with vehicle or proteasome inhibitor MG132 (at 10 or 25 μM) for 1 hour before treatment with vehicle or 1 μM FICZ for an additional 24 or 72 hours. MG132 was added every 24 hours in the 72-hour samples. Cell lysates were collected and analyzed by Western blotting for AHR and β-tubulin (loading control). Full length blots are shown in Supplemental Fig. 19).

To complement our genetic approach, we also tested the specific small molecule inhibitor of AHR, CH-223191^26^. Here, GOFs were plated and treated with vehicle or CH-223191 (at 1 or 10 μM) for 1 hour before treatment with vehicle or 1 μM FICZ for an additional 24 or 72 hours. CH-223191 was added every 24 hours in the 72-hour samples. Cell lysates were collected and analyzed by Western blotting for MMP1 (Fig. 7a). CH-223191 alone did not significantly alter MMP1 expression, however, at the 24-hour timepoint, 10 μM CH-223191 attenuated FICZ-induced MMP1 expression. At 72 hours, both 1 and 10 μM CH-223191 attenuated FICZ-induced MMP1 expression. Taken together, these data show that AHR is required for FICZ-induced MMP1 expression and activity in orbital fibroblasts.

AHR is degraded by the proteasome following ligand activation^28^. Consistent with this, OF AHR expression is dramatically reduced by 1 μM FICZ after 72 hours of treatment, see Fig. 6a, Supp Figs. 8–12 and our previous work^23^. To determine if proteasomal degradation requires ligand binding to AHR, GOFs were plated and treated with FICZ and/or CH-221391 as above for 24 or 72 hours. Cell lysates were collected and analyzed by Western blotting for AHR (Fig. 7b). FICZ treatment reduced AHR expression by 70–80% of baseline levels at 24 and 72 hours. CH-223191 alone did not significantly alter AHR expression. At the 24-hour timepoint, 1 or 10 μM CH-223191 slightly attenuated FICZ-induced AHR degradation (from 20% of vehicle levels to 30% of vehicle levels). At 72 hours, both 1 and 10 μM CH-223191 attenuated FICZ-induced AHR degradation (from 30% of vehicle levels to 40–50% of vehicle levels). While CH-223191 moderately attenuates FICZ-induced AHR protein degradation, a more direct assay is to block the proteasome with a small molecule inhibitor such as MG132^28^. Thus, to confirm that FICZ induces proteasomal degradation of AHR, GOFs were treated with MG132 (at 10 or 25 μM) with or without FICZ activation for 24 or 72 hours (Fig. 7c). Cell lysates were collected and analyzed by Western blotting for AHR. Again, FICZ lead to a dramatic reduction in AHR levels at both 24 and 72 hours. MG132 alone had little effect on AHR protein levels. However, when both FICZ and MG132 were added to the cells, MG132 completely blocked the ability of FICZ to induce AHR protein degradation (at both 24 and 72 hours). This data reveals that FICZ induces AHR ligand-dependent proteasomal degradation in GOFs at both 24 and 72-hour timepoints.

**Figure 8:**
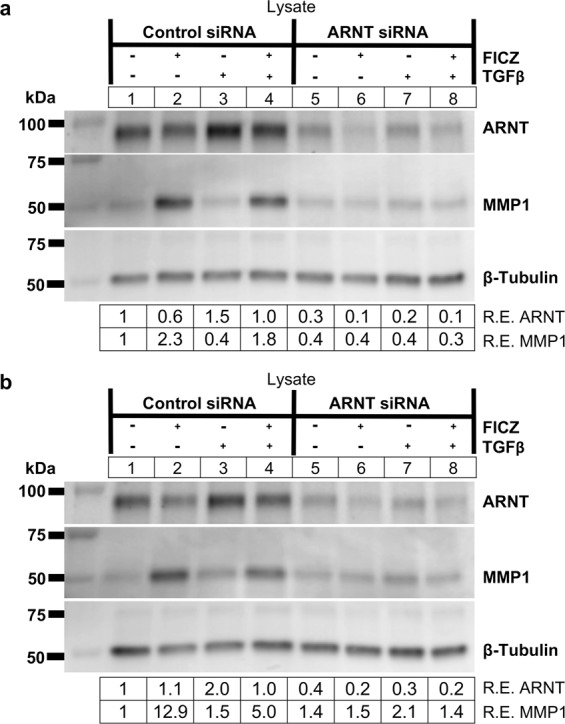
FICZ-induced MMP1 expression occurs in an ARNT-dependent manner. GOFs were treated with control or *ARNT* specific siRNA for 48 hours and then treated with either vehicle (DMSO) or the AHR ligand FICZ (1 µM), and TGFβ (5 ng/mL) as indicated. **(a)** After 72 hours of TGFβ treatment, cell extracts were isolated and analyzed by Western blot for ARNT, MMP1, and β-tubulin (loading control). Relative protein expression (R.E.) based on densitometry are listed below the images. *ARNT* siRNA reduced ARNT protein expression to 30 - 40% of control siRNA levels. Depletion of ARNT by *ARNT* siRNA blocks FICZ mediated induction of MMP1 expression. **(b)** An additional GOF strain is shown with the same setup as in **(a)**. The experiment was also performed in a NOF strain and is presented in Supplemental Fig. 13. Full length blots for this figure are located in Supplemental Fig. 20. Full length blots for Supplemental Fig. 13 are found in Supplemental Fig. 32.

AHR forms a heterodimer with ARNT to activate transcription of AHR-dependent genes^29,30^, therefore the requirement for ARNT during FICZ induced MMP1 expression was tested. GOF strains were treated with control or *ARNT* specific siRNA for 48 hours before treatment with vehicle or 1 μM FICZ +/− TGFβ for an additional 72 hours (Fig. 8). Cell lysates were collected and analyzed by Western blotting. In both strains tested, *ARNT* siRNA resulted in a greater than 60% depletion of ARNT protein levels compared to control siRNA (Fig. 8a,b). MMP1 levels were not increased by FICZ in *ARNT* siRNA treated cells whereas in control siRNA treated cells, MMP1 levels were induced 2 to 12-fold over vehicle (Fig. 8a,b). These results show that both AHR and ARNT are required for FICZ induced MMP1 expression.

## Discussion

**Figure 9:**
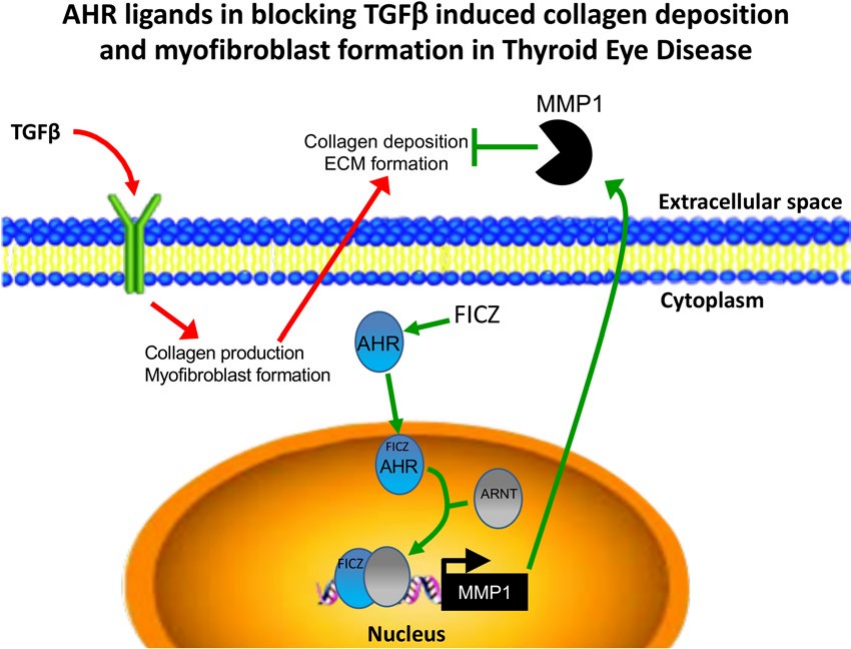
Summary of the role of AHR ligands in blocking TGFβ induced collagen deposition and myofibroblast formation in Thyroid eye disease. TGFβ signaling induces collagen 1 expression and myofibroblast formation in orbital fibroblasts. However, in the presence of an AHR ligand like FICZ, AHR binds to its ligand and then translocates to the nucleus. There, it heterodimerizes with its binding partner, ARNT. This complex activates transcription of the interstitial collagenase, MMP1. MMP1 is secreted into the extracellular matrix and degrades fibrous collagen in the extracellular space of the orbit.

Here, we show that MMP1 expression is regulated by the AHR pathway in human orbital fibroblasts. First, we determined that the tryptophan derivative and natural AHR ligand, FICZ induces *MMP1* mRNA and protein expression in OFs. Secondly, the induction of MMP1 expression by FICZ requires both the AHR and ARNT, suggesting that the AHR-ARNT transcriptional complex is required for MMP1 production in human OFs. Finally, AHR induced MMP1 reduces TGFβ-dependent collagen 1 accumulation to attenuate unwanted ECM production by activated orbital fibroblasts. Based on this, we present a model whereby AHR binds FICZ, promoting AHR translocation into the nucleus and then heterodimerizes with ARNT to induce MMP1 expression. In turn, MMP1 is transported to the extracellular matrix where the metalloproteinase catalyzes the degradation of TGFβ-driven collagen fibers (Fig. 9). Taken together, these studies suggest that AHR activation by FICZ is a promising therapeutic approach that may be able to block detrimental ECM production and tissue remodeling that occurs in TED.

We previously showed that FICZ could block OF proliferation and myofibroblast differentiation^23^. Furthermore, the ability of FICZ to prevent myofibroblast formation required AHR expression. Now, we show that FICZ increases orbital fibroblast expression of MMP1, an interstitial collagenase that degrades collagen I fibers. These data suggest that increased MMP1 levels may help reverse established ECM in TED orbits. This concept is especially appealing given the chronic feed forward loop of inflammation and OF activation in TED. Infiltrating T cells, macrophages and mast cells produce pro-inflammatory cytokines such as IL-1β and the pro-scarring cytokine, TGFβ to activate orbital fibroblasts^25^. Once activated, OFs produce high levels of hyaluronan and collagen that lead to edema and stiffening of the orbit^3,31^. The cycle continues as infiltrating T cells are retained and activated by excessive collagen I fibers, which can stimulate retroorbital and peripheral blood T cell proliferation and activation^32^. Therefore, FICZ induced MMP1 may increase clearance of T cells, preventing the cycle of inflammation and orbital connective tissue remodeling.

The ability of FICZ to increase MMP1 levels and thus limit inflammation and fibrotic ECM buildup in TED may have implications for a wide range of inflammatory and autoimmune diseases. Indeed, others have demonstrated the ability of FICZ to limit severity of inflammatory signaling in animal models of disease^16^. For example, in a mouse model of atopic dermatitis, topical treatment with FICZ reduces inflammatory cytokine production and mast cell accumulation compared to control^33^. Another study showed that in a mouse peritonitis model, FICZ reduced inflammasome activation and reduced IL6, IL-1β and TNFα levels^34^. Additionally, in a mouse model of colitis, FICZ reduced TNFα and IFNγ expression as well as attenuating weight loss and colitis symptoms^35^. These studies did not look directly at the contribution of decreased collagen levels and/or increased MMP1 in promoting these effects; however, future studies addressing these mechanisms could prove insightful.

FICZ is a high affinity ligand for AHR. However, some effects of FICZ may be AHR independent. FICZ accelerates wound healing, keratinocyte migration and proliferation even in the presence of *AHR* siRNA or a specific AHR antagonist^36^. In that study, FICZ promoted ERK signaling independently of AHR to increase keratinocyte growth. Our studies suggest that FICZ blocks orbital myofibroblast proliferation^23^ and increases MMP1 production to limit collagen in an AHR dependent manner (Figs. 1 and 6). Additionally, we now show that ARNT is required for FICZ induced MMP1 expression (Fig. 8). Since both AHR and ARNT are essential for FICZ induced MMP1 production in OFs, our data suggest that the AHR-ARNT transcriptional complex is activated. This concept is further supported by our reporter gene studies showing that the MMP1 promoter is responsive to FICZ in OFs (Fig. 2).

Directly blocking the 26 S proteasome with MG132 completely blocked FICZ-induced AHR protein degradation (Fig. 7c). This is consistent with previous reports showing that AHR protein is degraded by the proteasome. Interestingly, AHR protein levels are also reduced by TGFβ treatment, by around 15% in vehicle treated cells and by 50% in TGFβ-FICZ vs FICZ alone treated cells (see results in Fig. 6b and Supp Figs. 8 and 12). This has important implications for TGFβ-AHR pathway crosstalk and suggests why FICZ-induced MMP1 expression is attenuated by TGFβ (see MMP1 blot in Fig. 6a). While we did not investigate the mechanism here, we hypothesize that activation of TGFβ promotes AHR degradation by the proteasome. Interestingly, several E3 ubiquitin ligases that promote specific protein degradation by the proteasome such as Kelch-like protein 42^37^ and arkadia^38^ are activated by the TGFβ pathway. Currently, it is unclear if these E3 ligases promote degradation of AHR, however future studies investigating this are warranted.

Compared to the robust effect of MG132, CH-223191 only moderately blocked FICZ-induced AHR protein degradation through the proteasome pathway (Fig. 7b). This difference is likely due to the affinities of FICZ and CH-223191 for AHR. FICZ binds to the AHR with one of the highest affinities of any known AHR ligand (Kd ~10 pM- 1 nM)^16^. While we attenuated FICZ-induced MMP1 protein expression using CH-223191, it was not as effective as AHR knockdown by siRNA (compare Fig. 7a to Fig. 6a). Further studies using lower FICZ concentrations could allow for a complete inhibition using CH-223191.

In contrast, in the MMP1 promoter reporter assay, CH-223191 was able to completely block FICZ-induced activity (Fig. 2d). This plasmid reporter construct contains only ~1.1 kb of the proximal MMP1 promoter region. While we looked for canonical AHR DNA binding elements (formally termed dioxin response elements or DREs), none were identified in this proximal promoter region. While this region appears key to the ability of AHR to activate MMP1 expression, other important regulatory regions outside of this proximal region may also be involved in controlling MMP1 levels in GOFs. Global chromatin immunoprecipitation (ChIP)-seq studies in TCDD treated MCF-7 cancer cells have identified that AHR binding sites can be either proximal to the transcriptional start site or many kilobases away from confirmed or putative target genes^39^. Earlier computational work identified thousands of putative DREs^40^ close to transcriptional start sites (a range of −5000 to +2000 bp) of human genes^41^. Using the supplementary data from this study, we found that the human MMP1 gene showed two relatively low scoring DREs at −1051 bp and +1015 bp. These DREs may be involved in regulating FICZ induced MMP1 expression in GOFs. Interestingly, around 50% of DNA regions enriched in AHR ChIP-seq data from TCDD treated hepatic tissue do not contain a consensus DRE^42^. Thus, other non-DRE regions, present in the proximal promoter region, may be important in activating MMP1 expression. Future studies will be required to identify if AHR is directly binding to one of these regions in GOFs. This could be tested using chromatin immunoprecipitation (ChIP) assays or *in vitro* with GOF nuclear extract based electrophoretic mobility shift assays (EMSA) using putative DREs or other, non-canonical regions from the MMP1 promoter as probes.

Given its essential role in the AHR-transcriptional activation complex, our data also suggest a critical role for ARNT in limiting inflammation and scarring in TED. ARNT, is also referred to as hypoxia-inducible factor-1β (HIF-1β), as it functions as a transcriptional partner to HIF-1α as well as AHR. Thus, ARNT plays a central role in the AHR-mediated xenobiotic response and the HIF-1α-mediated response to hypoxia. This pathway crosstalk has led to the hypothesis that AHR and hypoxia pathways compete for a limiting supply of factors^43^. For example, using pathway specific reporters, it was found that expression of the prototypical AHR responsive gene, CYP1A1, is reduced when hypoxia is activated^43^. Other studies have subsequently shown that hypoxia and AHR related pathways may be in competition with each other based on limiting levels of ARNT^44,45^. However, while several studies have hypothesized about AHR- HIF-1α competition, it is still a matter of debate in the field. Due to tissue remodeling and the elevated inflammatory state, the orbit of TED patients is likely a low oxygen or hypoxic environment. Therefore, many aspects of TED pathology may be driven by HIF-1α. In hypoxia, HIF-1α is increased and subsequently, AHR transcriptional activity may be reduced. We hypothesize that in a hypoxic orbital space, ARNT mediates HIF-1α responses over AHR, leading to a loss of MMP1 expression, increased collagen accumulation and tissue remodeling. One of the highest risk factors for TED is smoking, which increases the chance of developing disease by over 7-fold^46,47^. One mechanism whereby smoking increases TED symptoms may be by promoting hypoxia and disrupting the balance between AHR and HIF-1α signaling. Future studies analyzing this competition in OFs may further reveal mechanism(s) driving TED pathophysiology.

Depending on their class and structure, MMPs degrade collagen, fibronectin, elastin, laminin, proteoglycans and others proteins in the ECM. MMP1 was the first collagenase characterized but other MMPs also target collagen^6^. MMP8 and MMP13 also degrade collagen I fibers; however, the cellular source and regulation of these MMPs are distinct. Infiltrating neutrophils are the predominant source of MMP8^48^ and MMP13 is most notably involved in degrading cartilage in osteoarthritis^49^. MMP2 and MMP9 degrade collagen IV fibers and thus control different aspects of the ECM. Our collagen zymogram results using collagen I yielded signals that derived from MMP1, as MMP1 depletion by specific siRNA dramatically reduced these signals (Fig. 5). However, we also observed a constitutive signal (Figs. 3–5) that is likely from MMP2. MMP2 is constitutively expressed by orbital fibroblasts (Figs. 1 and 5) and has been shown to possess collagenase I activity *in vitro* in previous studies. Furthermore, recombinant MMP2 gave a positive signal in our zymograms well (Supp Fig. 7). While MMP2 appears to have collagenase activity in these zymography assays, it does not appear to target COL1A1 in orbital fibroblasts, as MMP1 does. This highlights the different roles of MMPs *in vivo* and also the need to determine the specificity of signal from zymography assays.

Regulation of MMP1 activity can also be controlled by tissue inhibitor of metalloproteinases (TIMPs). TIMP1 is a glycoprotein inhibitor of MMP1 activity. While we did not study TIMP1 expression here, future studies looking into the role of TIMP1 in collagen accumulation and MMP1 activity in TED could provide a more complete understanding of the regulation of MMP1 in orbital fibroblasts. Studies in gingival fibroblasts have shown that over-expression of TIMP1 results in collagen over-expression^50^. Additionally, TGFβ can induce expression of TIMP1 in some fibroblasts and thus is another mechanism whereby TGFβ may reduce MMP1 activity to promote collagen accumulation^51^.

Our data show a robust FICZ-mediated increase in expression of MMP1 in OFs from both non-TED and TED patients. While GOFs have increased inflammatory activation, elevated proliferation rates and increased responses to TGFβ compared to NOFs, their ability to respond therapeutically to AHR activation is similar^7,52^. Therefore, the ability of AHR activation to control MMP1 activity is independent of disease. Thus, aspects of AHR-related therapeutic potential (AHR ligands block myofibroblast and ECM formation) are a generalizable phenomenon, thereby increasing the impact and scope of this work. Indeed, others have shown that AHR plays a role in controlling MMP1 production in scleroderma and periodontal ligament cells^53,54^.

New therapies are needed to block the detrimental extraocular muscle scarring and soft orbital connective tissue remodeling observed in TED. The data presented herein reveal that activation of the AHR by FICZ increases MMP1 levels leading to a reduction in collagen levels. Other AHR ligands that are already in use clinically may function in a similar manner. We recently showed that the proton pump inhibitors, esomeprazole and lansoprazole are AHR ligands that block orbital fibroblast to myofibroblast differentiation^24^. While we did not test the ability of PPIs to induce MMP1 expression in orbital fibroblasts, a recent study showed that patients on PPIs have elevated levels of MMP1 in gastric corpus biopsies^55^. Given these data, it may be interesting to perform a retrospective study on TED patients who also receive PPIs to determine if symptoms or outcomes are improved. However, before FICZ or other putative AHR ligands can be used clinically in TED patients, additional studies including pre-clinical animal models are essential.

## Experimental Procedures

### Cell culture

Primary human OFs were isolated and cultured using previously established explant techniques from either TED patients undergoing orbital decompression surgery (herein referred to as Graves’ orbital fibroblasts (GOFs) or normal orbital fibroblasts from patients undergoing unrelated orbital surgery (herein referred to as NOFs) at the Flaum Eye Institute^56^. The non-TED patients did not have any inflammatory orbital diseases. Each fibroblast strain used in this study came from a distinct individual and fibroblast strains were characterized as previously described^57^. Tissue procurement procedures followed the tenets of the Declaration of Helsinki and were approved by the University of Rochester Medical School Research Subjects Review Board. Informed, written consent was obtained from all patients before surgeries. NOFs and GOFs were cultured in Dulbecco’s modified Eagle’s Medium (DMEM) supplemented with 10% fetal bovine serum (FBS) and antibiotics. All media and supplements were purchased from Gibco (Carlsbad, CA) or Corning Life Sciences (Corning, NY). FBS was from Hyclone (Logan, UT). Orbital fibroblasts were placed in 0.1% FBS-DMEM for 48–72 hours before addition of 5 ng/ml TGFβ (R&D Systems, Minneapolis, MN) for an additional 24–72 hours to drive formation of myofibroblasts. The AHR ligand 6-formylindolo[3,2-b]carbazole (FICZ) was obtained from Enzo Life Sciences (Farmingdale, NY) and was added at concentrations as described to selected samples 1–2 hours before addition of TGFβ. The inhibition of AHR was accomplished by using the compound CH-223191 from Sigma-Aldrich (St. Louis, MO) at concentrations indicated, both in the presence and absence of FICZ, for 24 and 72 hours. MG132 (Calbiochem, Temecula, CA) was used at 10 µM and 25 µM to inhibit the proteasome.

### Gene expression knockdown using siRNA

*AHR* siRNA (siRNA ID number: s1199), *ARNT* siRNA (siRNA ID number: s1613) and *MMP1* siRNA (siRNA ID number: s8847) were purchased from Ambion’s Silencer Select pre-designed siRNA library (Grand Island, NY). A non-specific, negative control siRNA (negative control #1, Ambion) was used as the control. Cells were grown to 70–80% confluence in 6-well plates and treated with the siRNAs mixed with Lipofectamine 2000 (Invitrogen, Carlsbad, CA) in OptiMEM I (Invitrogen, Carlsbad, CA) at a final concentration of 50 nM for 24 hours, according to the manufacturers’ instructions. Cells were then treated with or without TGFβ and/or FICZ as described.

### Western blot analysis

Supernatants were collected and concentrated using Amicon Ultra 10 K Centrifugal Filter Devices (Millipore, Billerica, MA). Cells were homogenized with lysis buffer containing 50 mM Tris-HCl pH 6.8, 2% sodium dodecyl sulfate and a protease inhibitor cocktail (Cell Signaling Biotechnology, Beverly, MA). Total protein concentration was determined with the detergent compatible (DC) protein detection assay (BioRad, Hercules, CA). 5 µg of protein for both lysates and supernatants were separated via SDS-PAGE, transferred onto 0.45 μm Immobilon-PVDF membranes (Millipore, Billerica, MA), and blocked with 5% non-fat dry milk and 0.1% Tween 20 (BioRad, Hercules, CA) in 1X TBS. The following antibodies were from Cell Signaling Biotechnology (Danvers, MA): AHR (rabbit anti-AHR; 1:1000, 83200), ARNT (rabbit anti-HIF-1β/ARNT (D28F3); 1:1000, 5537), MMP2 (rabbit anti-MMP-2 (D2O4T); 1:1000, 87809), and β-tubulin (rabbit anti-β-tubulin; 1:1000, 2146). Antibodies targeting MMP1 (mouse anti-MMP1; 1:1000, MAB901, R&D Systems, Minneapolis, MN), collagen 1 (goat anti-COL1A1; 1:200, sc-8783, Santa Cruz Biotechnologies, Santa Cruz, CA) and αSMA (mouse anti-αSMA; 1:6000, A2547, Sigma-Aldrich, St. Louis, MO) were used also used in Western blot experiments. Anti-mouse, anti-goat or anti-rabbit HRP-conjugated secondary antibodies were obtained from Jackson Immunoresearch (West Grove, PA). Protein was visualized using Immobilon Western chemiluminescent horseradish peroxidase substrate (Millipore, Billerica, MA). Chemiluminescent signals were captured using a VersaDoc MP imaging system (BioRad). Densitometric analysis was performed with Image Lab analysis software (BioRad). Ponceau S (Sigma-Aldrich) and/or Coomassie Blue (GE Healthcare, Pittsburg, PA) protein stains were used to confirm equal protein loading.

### Collagen zymography

To detect MMP1 activity in conditioned media, cell culture supernatants were collected from OFs that had been treated with vehicle, AHR ligand (FICZ) and/or 5 ng/ml TGFβ and concentrated as described above. Substrate zymography was performed as described previously^58^. Briefly, polyacrylamide gels were incorporated with 1 mg/ml collagen I from calf skin (Sigma-Aldrich) and an equal volume of samples were run under non-reducing conditions. Purified standards of MMP1 and MMP2 were purchased from Millipore Sigma (Danvers, MA). Gels were washed in 2.5% Triton X-100 for two hours at 37 °C with constant mechanical rotation. Gels were then briefly rinsed in distilled water and incubated in MMP1 incubation buffer (50 mM Tris-HCl, pH 7.4, 5 mM CaCl_2_, 0.001% NaN_3_, and 0.005% Triton X-100) for 36 hours at 37 °C with constant agitation. Gels were stained with 0.1% Coomassie Blue and destained in 6:3:1 dH_2_O:methanol:acetic acid. Collagenase activity was evident as clear (unstained) bands. Images were captured using a Gel Doc EZ Gel Documentation System (BioRad). Densitometric analysis was performed on inverted images with Image Lab analysis software (BioRad).

### RNA extraction

Total cell RNA was extracted using Qiazol lysis reagent (Qiagen, Valencia, CA) and isolated with a RNeasy Mini Kit according to the manufacturers’ instructions. Total RNA concentrations were determined with a NanoDrop 1000 spectrophotometer (Thermo Fisher Scientific, Wilmington, DE).

### Quantitative PCR (qPCR)

cDNA was generated using the iScript reverse transcription kit (BioRad) and gene expression quantified via real-time PCR with gene specific primers, a universal SsoFast Evergreen PCR master mix (BioRad) and an iCycler iQ5 PCR thermal cycler (BioRad). Gene specific primers are as follows: MMP1 (MMP1 fwd 5-CCCACAATGTCCCCATCTATG-3′) and (MMP1 rev 5′- TGAACAGCCCAGTACTTATTCC -3′), AHRR (AHRR fwd 5′- AGACGGATGTAATGCACCAG -3′) and (AHRR rev 5′- CCTCCCCAGGATAGCATCA -3′), CYP1A1 (CYP1A1 fwd 5′- CTTCGTCCCCTTCACCATC -3′) and (CYP1A1 rev 5′- TCAGGTAGGAACTCAGATGGG -3′) and CYP1B1 (CYP1B1 fwd 5′- CTATCACTGACATCTTCGGCG -3′) and (CYP1B1 rev 5′- CATACAAGGCAGACGGTCC - 3′), 18 S rRNA (18 S fwd 5′- GCACAAATCCCTTCTACCCG -3′) and (18 S rev 5′-ACTACGAGCTTTTTAACTGC-3′).

### Luminex assay

*AHR* siRNA was used to knock down gene expression as described above. After cells had been treated with vehicle, 0.1 µM or 1 µm FICZ and/or 5 ng/ml TGFβ for 72 hours, conditioned media supernatants were collected. Supernatants were analyzed with a multiplex bead sandwich ELISA panel (Millipore Sigma, Burlington, MA) that contained antibodies against MMP1, MMP2 and MMP9. Cell culture media (50 μl) from triplicate wells were assayed to measure production of MMP1, MMP2, and MMP9 using a Luminex FlexMAP 3D instrument following the manufacturer instructions. MMP levels are reported as pg/ml.

### Luciferase reporter assay

Plasmid DNA was introduced into orbital fibroblasts seeded in 6 well plates using Lipofectamine 2000 (Invitrogen) following the manufacturer’s suggested protocol. The MMP1-promoter-Renilla luciferase reporter construct was obtained from SwitchGear Genomics (Carlsbad, CA). The construct contains a 1,177 bp insert upstream of the Renilla luciferase open reading frame that corresponds to the human MMP1 promoter region (−1030 to +146). A control construct without the MMP1 promoter (SV40 promoter-Renilla luciferase construct) was also used. Fibroblasts were treated with vehicle (DMSO), 0.1 or 1.0 μM FICZ for 16 hours after transfection. For some samples, CH-223191 (10 μM) was added 30 minutes before addition of FICZ. Following incubation, cell culture medium was removed and cells lysed directly in plates using Renilla Glo assay reagent (Thermo Fisher, Waltham, MA). Renilla luciferase readings were measured on a Varioskan Flash luminescent plate reader (Thermo Fisher). Luciferase readings were normalized to total protein and are reported as normalized luciferase activity based on luminescent signal of vehicle treated cells.

### Statistical analysis

All experiments were repeated at least three times using different strains of orbital fibroblasts with biological replicates (n = 3 or more) to drive the experiments. Student’s t-test and one-way or two-way analysis of variance (ANOVA) were used for statistical analysis and p-values of p < 0.05; p< 0.01; p< 0.001; were considered significant.

## Supplementary information

Supplementary information.
