# Supplementary material for: The aryl hydrocarbon receptor pathway controls matrix metalloproteinase-1 and collagen levels in human orbital fibroblasts
Source: Sci Rep. 2020 May 21;10:8477. doi: 10.1038/s41598-020-65414-1 (PMC7242326; doi:10.1038/s41598-020-65414-1)
Supplement: Supplementary file 1 — Supplementary information. [file 41598_2020_65414_MOESM1_ESM.pdf]

Supplementary Information

The aryl hydrocarbon receptor pathway controls matrix metalloproteinase-1 and collagen levels in human orbital fibroblasts

Elisa Roztocil[1], Christine Hammond[1], Mithra O. Gonzalez[1], Steven E. Feldon[1] and Collynn F. Woeller[1,2*]

From the [1]Flaum Eye Institute, [2]Department of Environmental Medicine School of Medicine and Dentistry, University of Rochester, Rochester, New York 14642, USA

Running title: AHR regulates MMP1 and collagen 1 in human orbital fibroblasts

[*]Corresponding author. EMAIL collynn_woeller@urmc.rochester.edu

Supplemental Figure 1

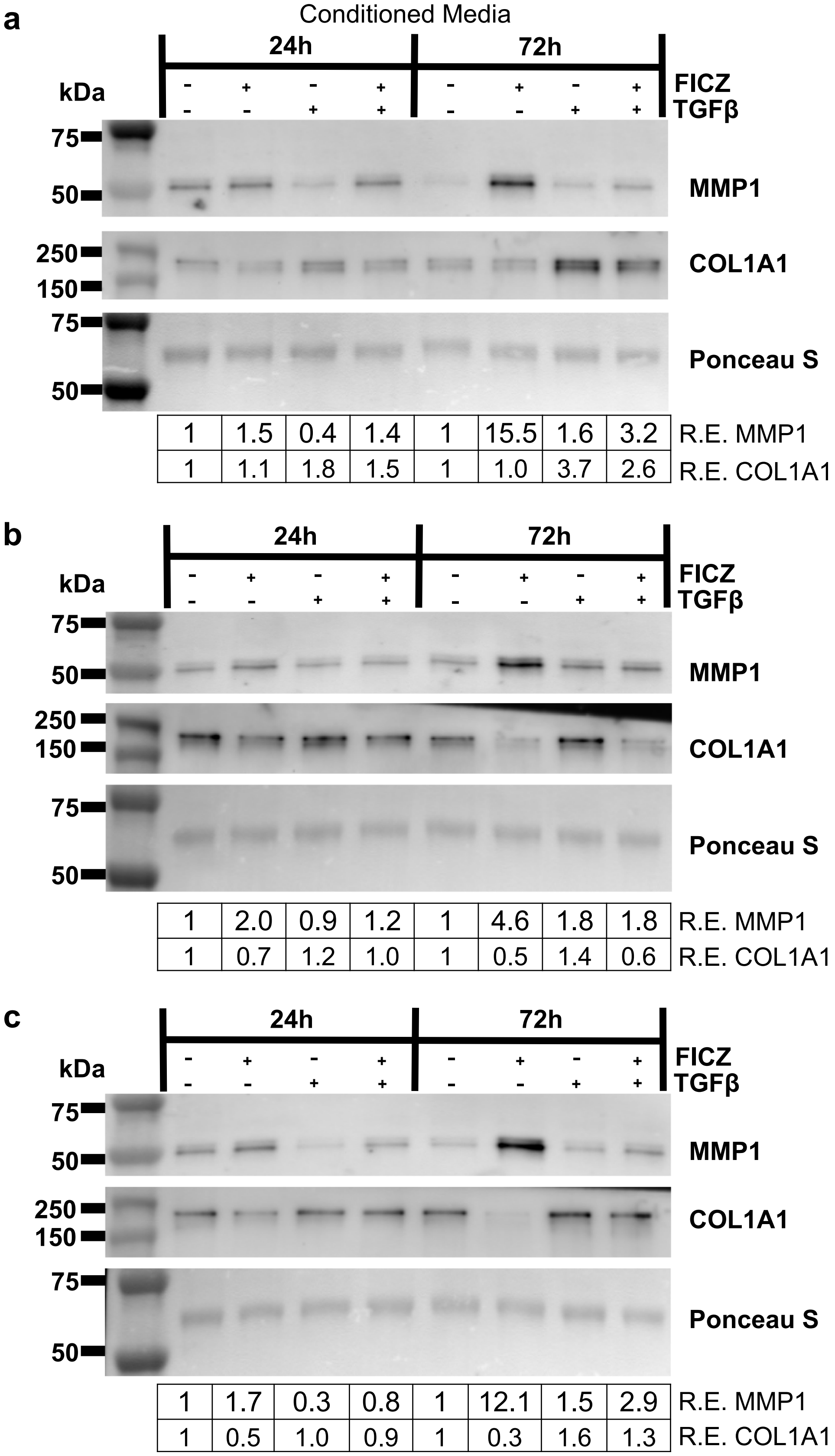

Supplemental Figure 1: The AHR ligand FICZ increases MMP1 secretion and decreases collagen production in TED orbital fibroblast strains. (a-c), TED orbital fibroblast strains were treated with FICZ with or without TGFβ for and 72 hours (each panel represents an individual strain). Conditioned media were collected and analyzed by Western blot. When GOFs are treated with the AHR ligand FICZ, MMP1 production increased. Full length blots can be seen in **Supplemental Figure 21**.

Supplemental Figure 2

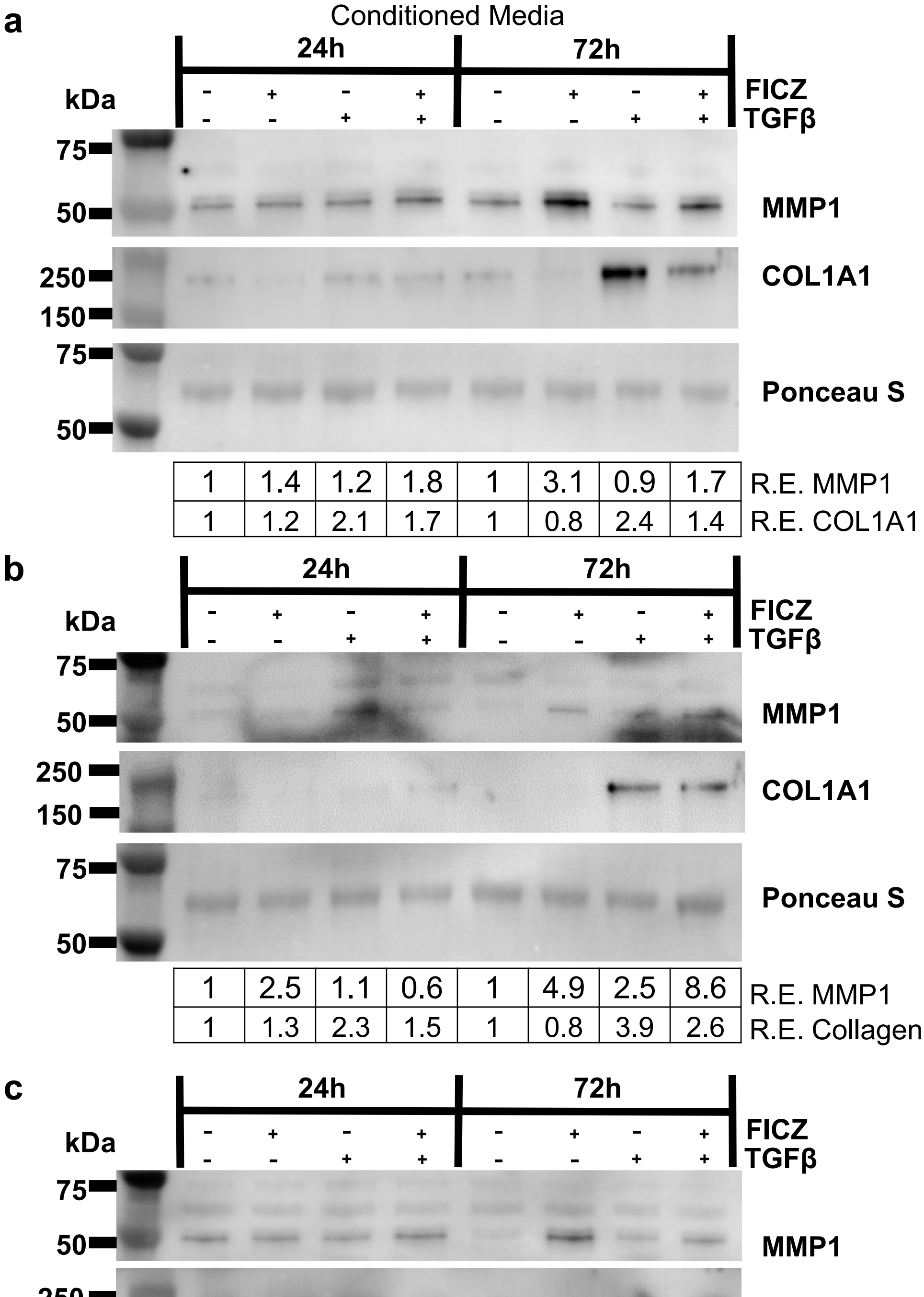

a

1	1.4	1.2	1.8	1	3.1	0.9	1.7	R.E. MMP1
1	1.2	2.1	1.7	1	0.8	2.4	1.4	R.E. COL1A1

b

1	2.5	1.1	0.6	1	4.9	2.5	8.6	R.E. MMP1
1	1.3	2.3	1.5	1	0.8	3.9	2.6	R.E. Collagen

c

1	1.4	1.0	1.8	1	7.1	2.2	3.3	R.E. MMP1
1	2.8	3.2	2.0	1	0.5	2.4	2.7	R.E. COL1A1

Supplemental Figure 2: The AHR ligand FICZ increases MMP1 secretion and decreases collagen production in non-TED orbital fibroblast strains. (a-c), Normal orbital fibroblast (NOF) strains were treated with FICZ with or without TGFβ for and 72 hours (each panel represents an individual strain). Conditioned media were collected and analyzed by Western blot. Like in GOFs, when NOFs are treated with the AHR ligand FICZ, MMP1 production increased. Full length blots for this figure are located in **Supplemental Figure 22**.

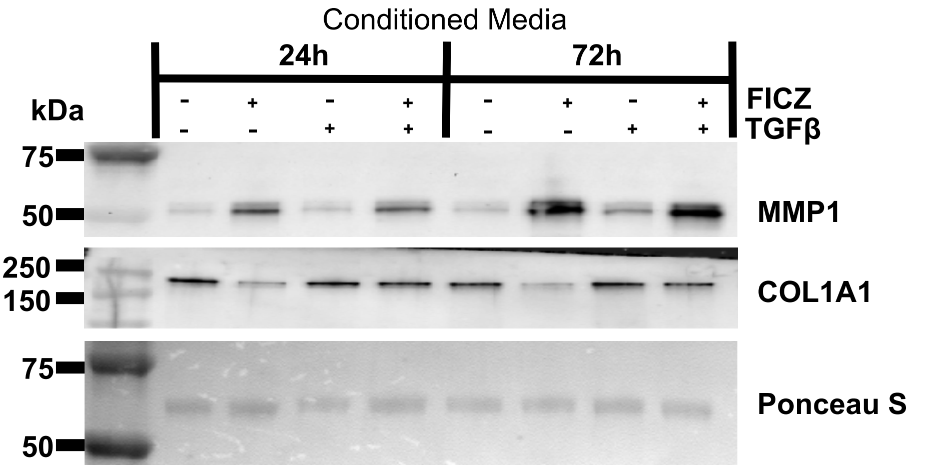

1	8.9	1.5	8.6	1	12.9	2.6	12.9	R.E. MMP1
1	0.3	0.9	0.9	1	0.4	1.3	0.8	R.E. COL1A1

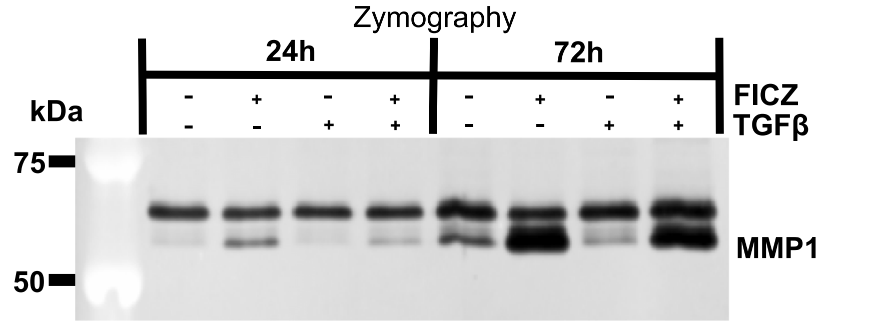

1	3.9	0.8	1.7	1	3.0	0.5	2.9	R.A. MMP1

Supplemental Figure 3: The AHR ligand FICZ increases MMP1 secretion and decreases collagen production in an AHR-dependent manner. An additional GOF strain was pretreated with FICZ, 1 µM, for 1-2 hours followed by 5 ng/mL TGFβ for an additional 24 and 72 hours. Conditioned media were collected and analyzed Western Blotting and collagen zymography. Upper panel, equal amounts of protein from culture supernatant were loaded. Relative protein expression (R.E.) based on densitometry is shown below each blot. Lower panel, the same supernatants were analyzed by zymography with collagen as substrate. Relative MMP1 activity (R.A.) based on densitometry of the inverted zymography gels is shown below each image. Importantly, only the lower band in the zymogram represents MMP1 activity. Full length blots can be seen in **Supplemental Figure 23**.

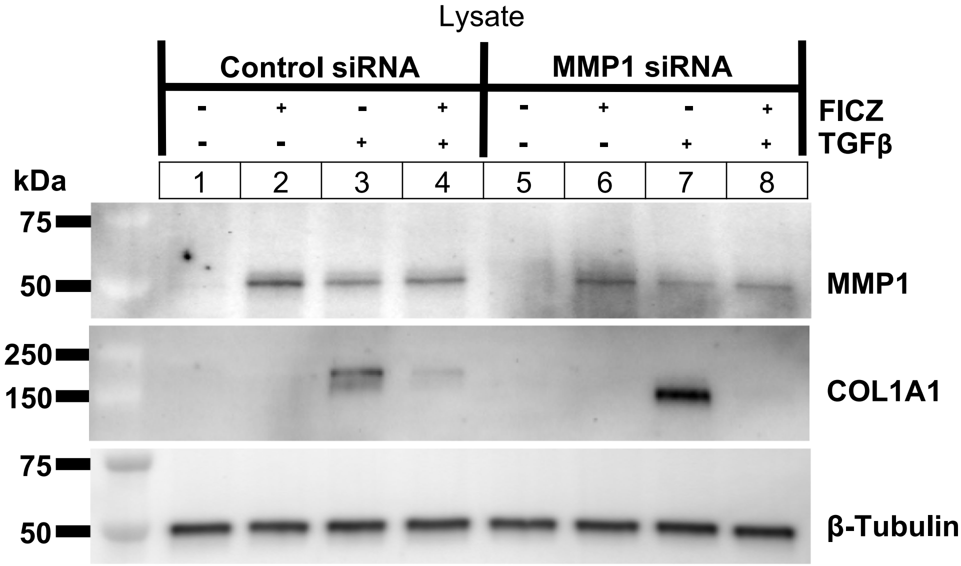

1	8.6	4.1	4.9	1.0	3.1	2.4	2.6	R.E. MMP1
1	0.8	5.8	2.0	1.6	2.8	12.6	3.5	R.E. COL1A1

Supplemental Figure 4: MMP1 knockdown attenuates FICZ mediated MMP1 production and increases collagen expression. GOFs were treated with control or *MMP1* specific siRNA for 48 hours and then treated with either vehicle (DMSO) or the AHR ligand FICZ (1 μM), and TGFβ (5 ng/mL) as indicated. After 72 hours of TGFβ treatment, cell extracts were isolated and analyzed by Western blot for MMP1, COL1A1, and β-tubulin (loading control). Relative protein expression (R.E.) based on densitometry are listed below the images. *MMP1* siRNA reduced MMP1 protein expression to less than 30% of control siRNA levels for treatments tested. COL1A1 increased in the presence of *MMP1* siRNA as compared to control siRNA. Full length blots are included in **Supplemental Figure 24**.

Supplemental Figure 5

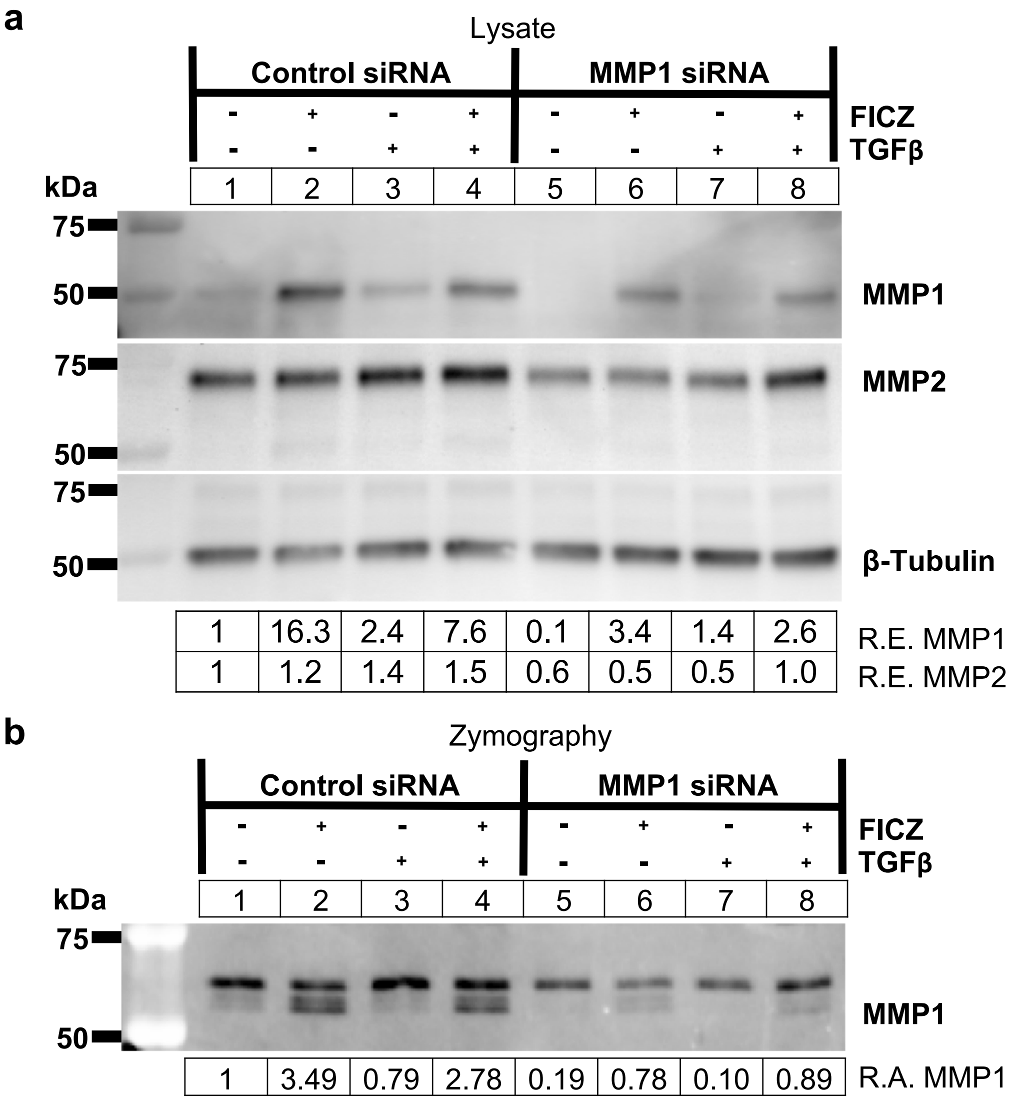

Supplemental Figure 5: MMP1 knockdown attenuates FICZ mediated MMP1 production and activity. GOFs were treated with control or *MMP1* specific siRNA for 48 hours and then treated with either vehicle (DMSO) or the AHR ligand FICZ (1 μM), and TGFβ (5 ng/mL) as indicated. **(a)** After 72 hours of TGFβ treatment, cell extracts were isolated and analyzed by Western blot for MMP1, MMP2, and β-tubulin (loading control). Relative protein expression (R.E.) based on densitometry are listed below the images. *MMP1* siRNA reduced MMP1 protein expression to less than 20% of control siRNA levels for all treatments tested. **(b)** Corresponding supernatants were also collected and analyzed using collagen I zymography. Relative MMP1 activity (R.A.) based on densitometry of the inverted zymography gel is shown below the image. The lower band, which represents MMP1 activity, is reduced more than 3-fold by *MMP1* siRNA. Full length blots for this figure are included in **Supplemental Figure 25**.

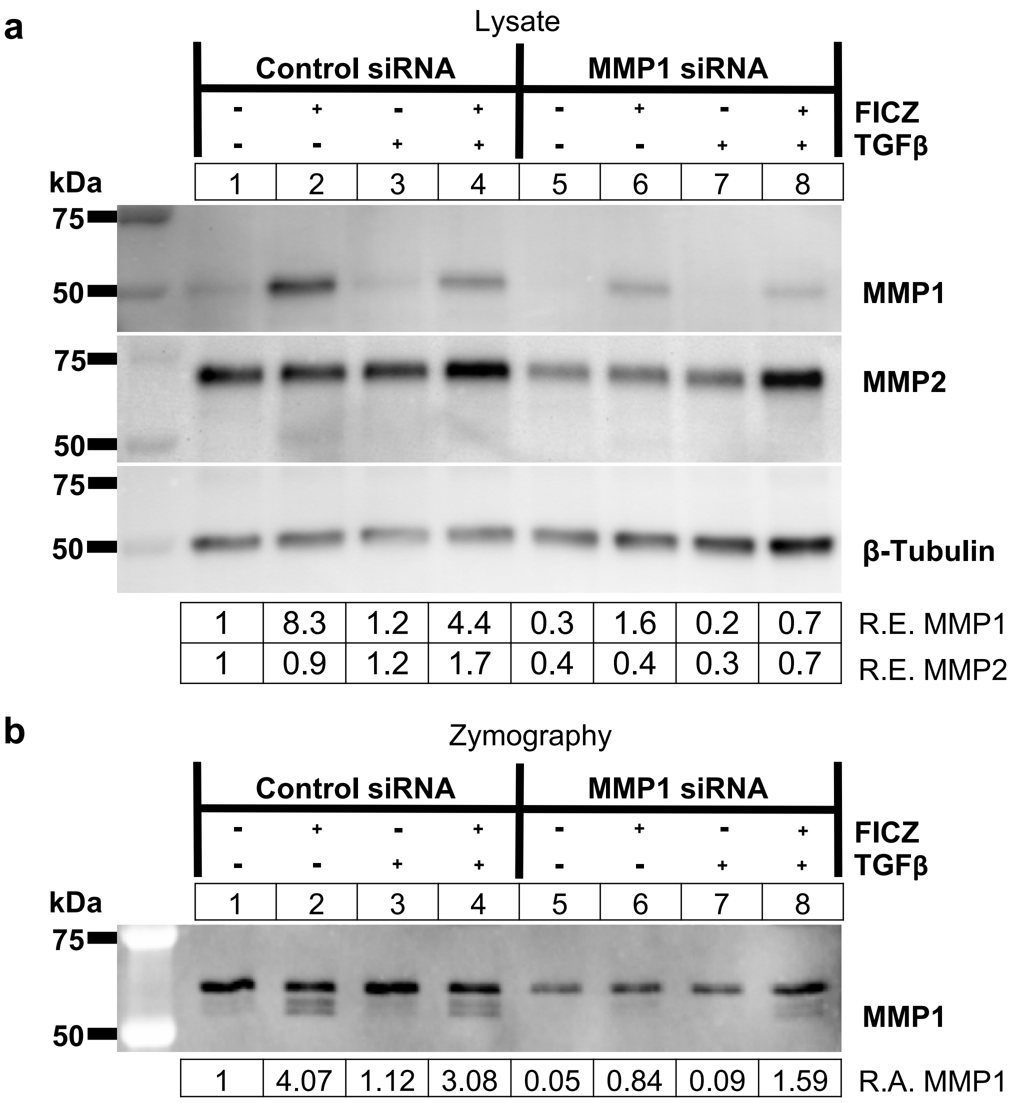

Supplemental Figure 6: MMP1 knockdown attenuates FICZ mediated MMP1 production and activity. NOFs were treated with control or MMP1 specific siRNA for 48 hours and then treated with either vehicle (DMSO) or the AHR ligand FICZ (1 µM), and TGFβ (5 ng/mL) as indicated. **(a)** After 72 hours of TGFβ treatment, cell extracts were isolated and analyzed by Western blot for MMP1, MMP2, and β-tubulin (loading control). Relative protein expression (R.E.) based on densitometry are listed below the images. *MMP1* siRNA reduced MMP1 protein expression to less than 15% of control siRNA levels. Corresponding supernatants were also collected and analyzed using collagen I zymography **(b)**. Relative MMP1 activity (R.A.) based on densitometry of the inverted zymography gel is shown below the image. The lower band, which represents MMP1 activity, is reduced more than 2-fold by *MMP1* siRNA. Full length blots are located in **Supplemental Figure 26**.

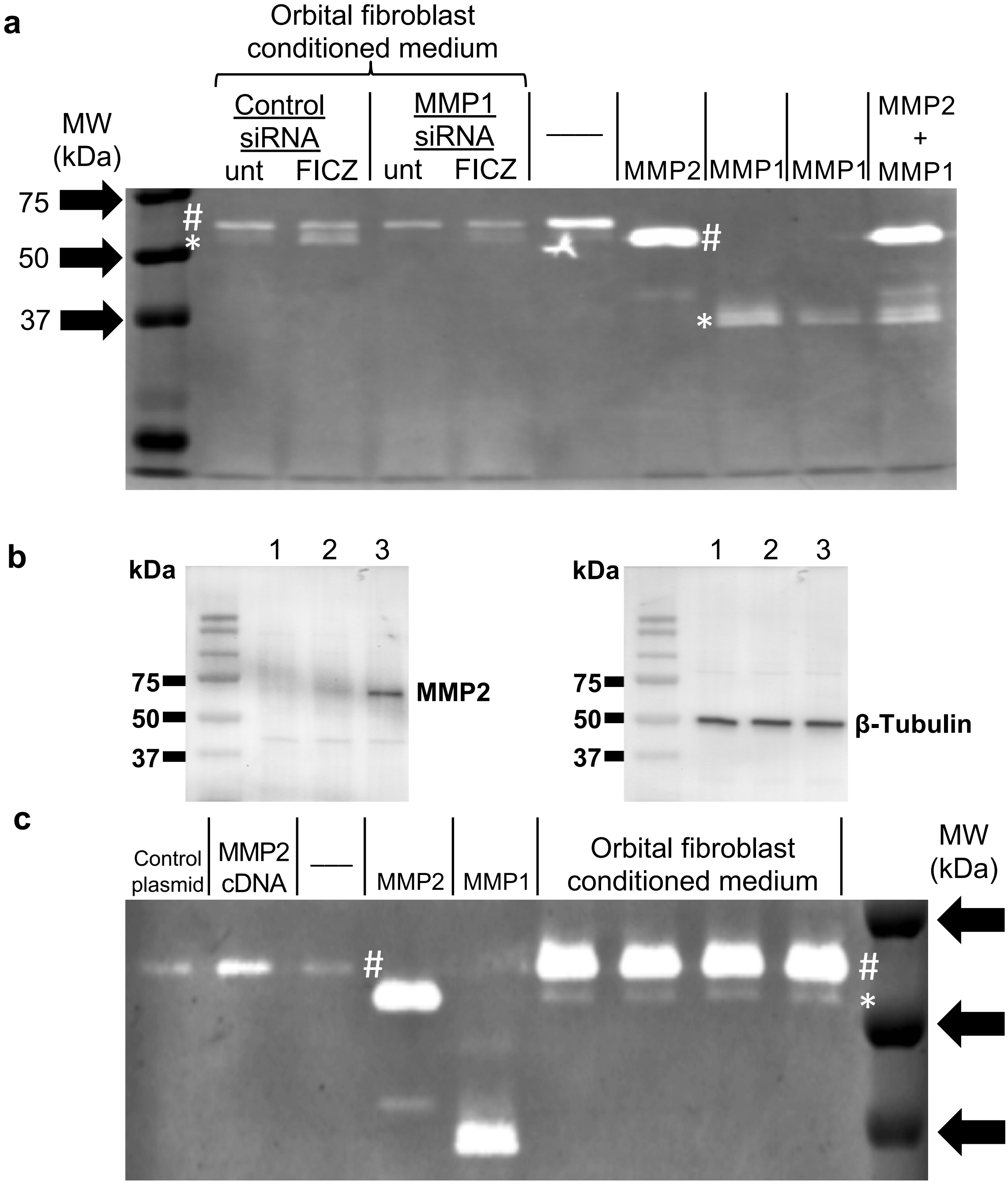

Supplemental Figure 7: Collagen gel zymography can be a substrate for MMP1 and MMP2.
(a) Collagen I gel zymography showing molecular weights and bands from orbital fibroblast supernatants and from recombinant protein standards. Depletion of MMP1 by *MMP1* siRNA reduces the MMP1 specific band (*) but not the MMP2 specific band (#). MMP standards run next to the samples show that both MMP2 and MMP1 can degrade collagen I in zymography gels. Note that purified MMP1 can undergo autocatalysis to cleave itself into smaller form, which is what is observed here. Additionally, recombinant MMP2 is also smaller than the observed MMP2 from orbital fibroblast medium. **(b)**Western blot of HEK293FT cells that had either a control plasmid, MMP1 cDNA plasmid (2) or MMP2 plasmid cDNA (3) introduced. **(c)** Zymography showing overexpression of MMP2 in 293FT cells increases observed MMP2 band in collagen zymography. The middle lanes show same recombinant MMP2 and MMP1 standards run in (**a**). Note that the MMP2 specific band in the 293FT medium (#) corresponds to the observed MW of MMP2 in the Western blot shown in (**b**) and to the MMP2 band of orbital fibroblast medium (#).

Supplemental Figure 8

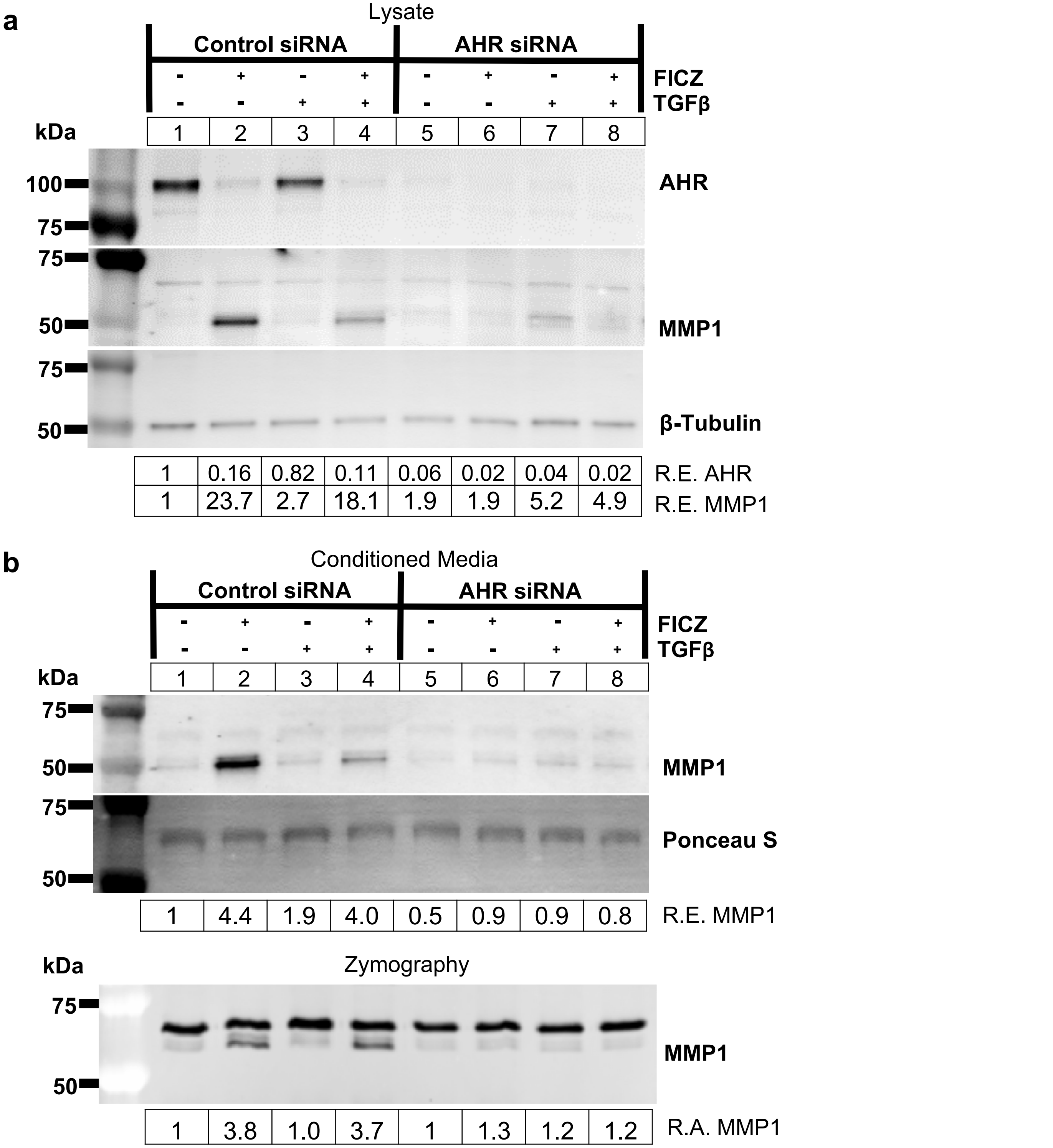

Supplemental Figure 8: FICZ-induced MMP1 expression and activity occurs in an AHR-dependent manner. GOFs were treated with control or *AHR* specific siRNA and treated as indicated. **(a)** AHR, MMP1 and β-tubulin (loading control) were analyzed by Western blot. **(b)** Corresponding supernatants were collected and analyzed using Western blotting. In the lower panel relative MMP1 activity (R.A.) based on zymography is shown. The lower band, which represents MMP1 activity, is reduced in FICZ treated samples by more than 10-fold by *AHR* siRNA. The top band, which corresponds to the molecular weight of MMP2, is not altered by *AHR* siRNA. Full length blots are included in **Supplemental Figure 27**.

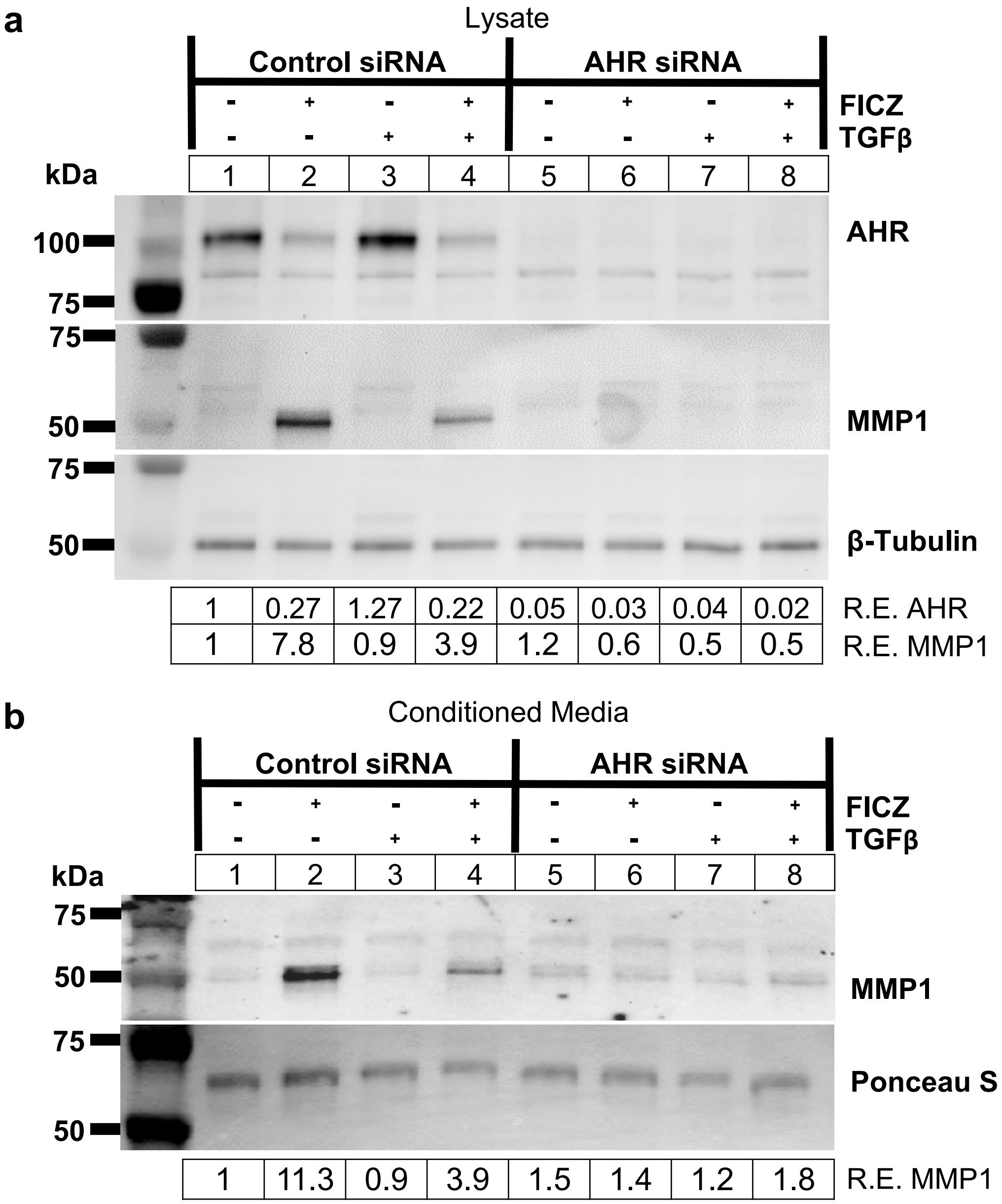

Supplemental Figure 9: FICZ-induced MMP1 expression and activity occurs in an AHR-dependent manner. GOFs were treated with control or *AHR* specific siRNA for 48 hours then treated with either vehicle (DMSO) or the AHR ligand FICZ (1 µM), and TGFβ (5 ng/mL) as indicated. **(a)** After 72 hours of TGFβ treatment, cell extracts were isolated and analyzed by Western blot for AHR, MMP1 and β-tubulin (loading control). Relative protein expression (R.E.) based on densitometry are listed below the images. *AHR* siRNA reduced AHR protein expression to less than 5% of control siRNA levels. **(b)** Corresponding supernatants were also collected and analyzed using Western blotting (upper panel) and collagen I zymography (lower panel). Relative MMP1 levels (R.E.) based on densitometry is shown below the blot images in the top panel. In the bottom panel, MMP1 activity (R.A.) based on densitometry of the inverted zymography gel is shown. The lower band, which represents MMP1 activity, is reduced in FICZ treated samples by more than 10-fold by *AHR* siRNA. The top band, which corresponds to the molecular weight of MMP2, is not altered by *AHR* siRNA. Full length blots for this strain are located in **Supplemental Figure 28**.

Supplemental Figure 10

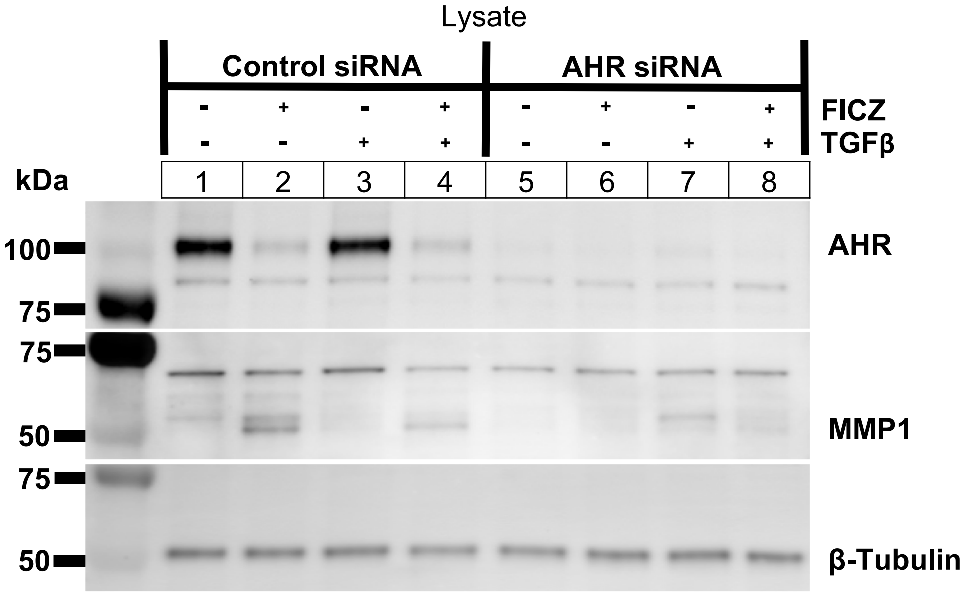

1	0.13	0.96	0.12	0.03	0.01	0.03	0.01	R.E. AHR
1	2.9	1.5	2.7	1.3	1.7	1.7	2.1	R.E. MMP1

Supplemental Figure 10: FICZ-induced MMP1 expression and activity occurs in an AHR-dependent manner. GOFs were treated with control or *AHR* specific siRNA for 48 hours then treated with either vehicle (DMSO) or the AHR ligand FICZ (1 μM), and TGFβ (5 ng/mL) as indicated. After 72 hours of TGFβ treatment, cell extracts were isolated and analyzed by Western blot for AHR, MMP1 and β-tubulin (loading control). Relative protein expression (R.E.) based on densitometry are listed below the images. *AHR* siRNA reduced AHR protein expression to less than 5% of control siRNA levels. *AHR* siRNA blocked the ability of FICZ to induce MMP1 expression. The full length blots for this figure are included in **Supplemental Figure 29**.

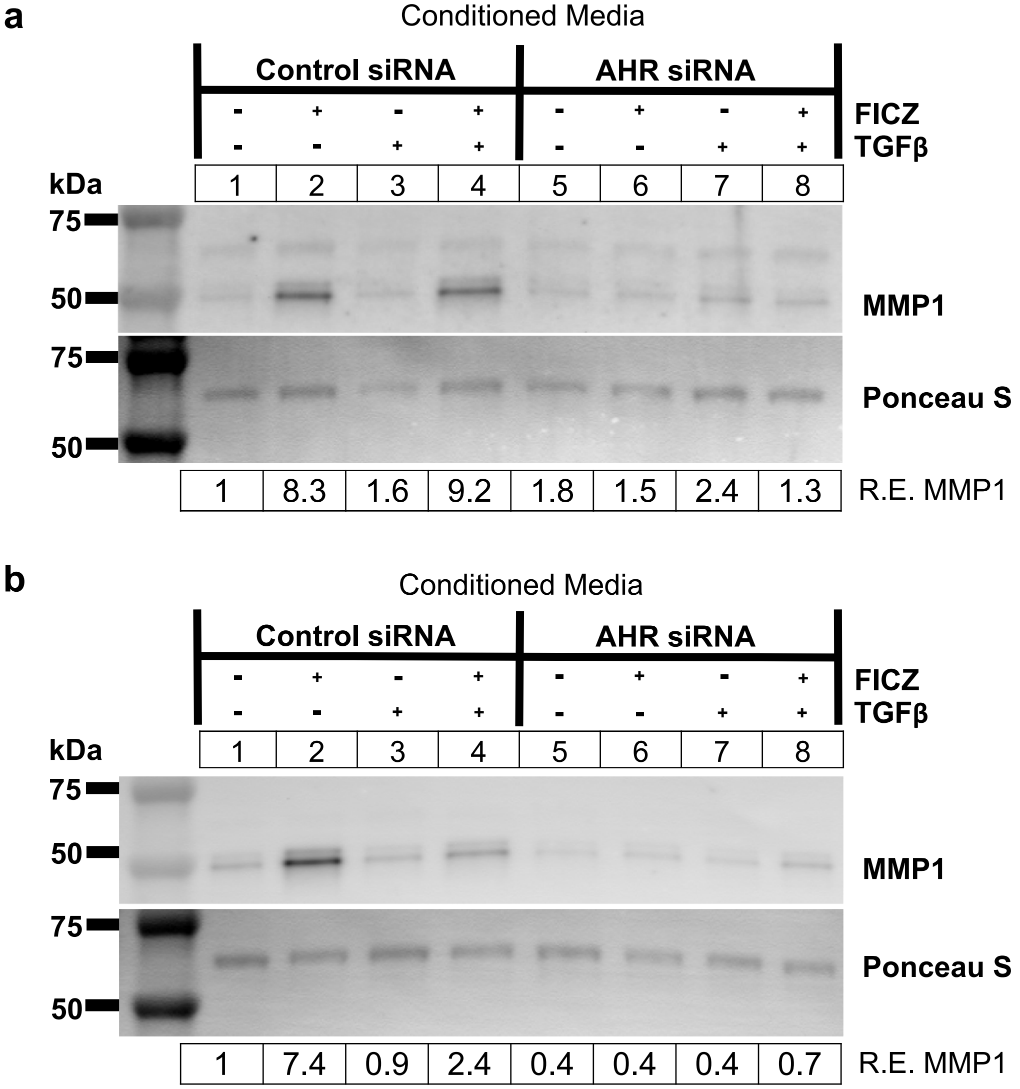

Supplemental Figure 11: FICZ-induced MMP1 expression and activity occurs in an AHR-dependent manner. (a) GOFs were treated with control or *AHR* specific siRNA for 48 hours then treated with either vehicle (DMSO) or the AHR ligand FICZ (1 μM), and TGFβ (5 ng/mL) as indicated. After 72 hours of TGFβ treatment, conditioned media was isolated and analyzed by Western blot for MMP1. Relative protein expression (R.E.) based on densitometry are listed below the images. *AHR* siRNA reduced AHR protein expression to less than 5% of control siRNA levels. *AHR* siRNA blocked the ability of FICZ to induce MMP1 expression. **(b)** A different strain was used and data collected as in **(a)**. Full length images for both strains can be seen in **Supplemental Figure 30**.

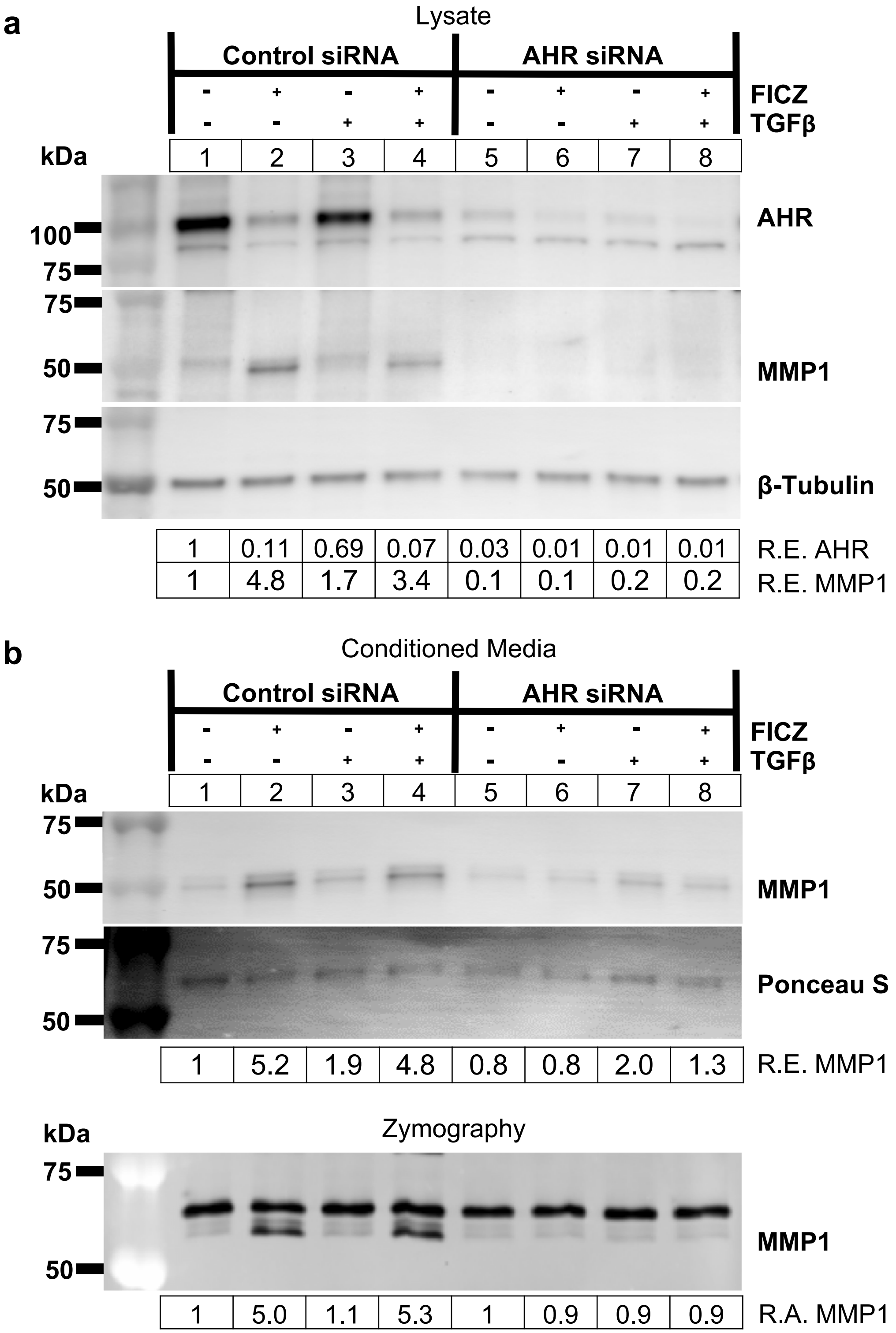

Supplemental Figure 12: FICZ-induced MMP1 expression and activity occurs in an AHR-dependent manner. NOFs were treated with control or *AHR* specific siRNA and treated as indicated. **(a)** AHR, MMP1 and β-tubulin (loading control) were analyzed by Western blot. **(b)** Corresponding supernatants were also collected and analyzed using Western blotting. Relative MMP1 activity (R.A.) based on zymography is also shown. The lower band, which represents MMP1 activity, is reduced in FICZ treated samples by more than 10-fold by *AHR* siRNA. The top band, which corresponds to the molecular weight of MMP2, is not altered by *AHR* siRNA. **Supplemental Figure 31** includes the full length blots for this figure.

Supplemental Figure 13

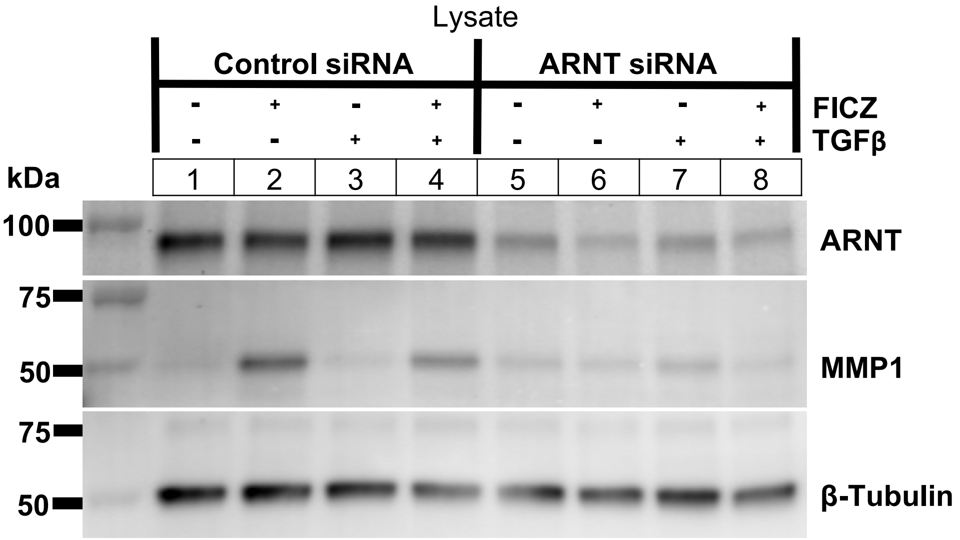

1	0.8	1.3	1.7	0.4	0.2	0.2	0.2	R.E. ARNT
1	7.2	0.8	7.7	1.8	1.2	1.9	1.1	R.E. MMP1

Supplemental Figure 13: FICZ-induced MMP1 expression and activity occurs in an ARNT-dependent manner. NOFs were treated with control or *ARNT* specific siRNA for 48 hours and then treated with either vehicle (DMSO) or the AHR ligand FICZ (1 μM), and TGFβ (5 ng/mL) as indicated. **(a)** After 72 hours of TGFβ treatment, cell extracts were isolated and analyzed by Western blot for ARNT, MMP1 and β-tubulin (loading control). Relative protein expression (R.E.) based on densitometry are listed below the images. *ARNT* siRNA reduced ARNT protein expression to less than 20% of control siRNA levels for all treatments tested. **(b)** An additional strain is shown with the same setup as in **(a)**. Depletion of ARNT by *ARNT* siRNA blocks FICZ mediated induction of MMP1 expression. The full length blots to accompany this figure are located in **Supplemental Figure 32**.

Supplemental Figure 14

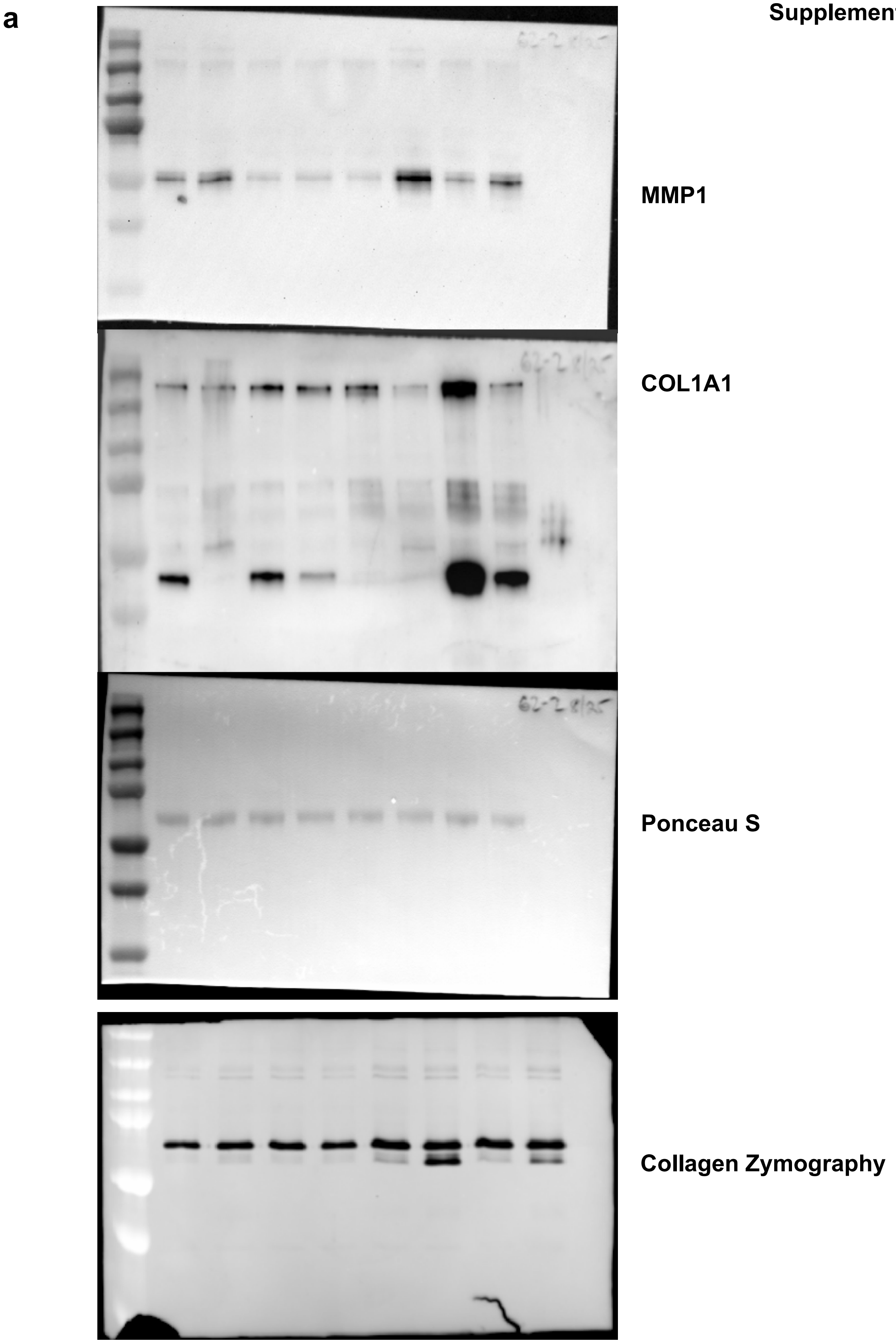

Supplemental Figure 14: Full length blots to accompany Figure 3A. Molecular weight marker is shown in the far left lane. The first 3 blot images are from the same membrane showing MMP1, Collagen 1A1 and total protein (Ponceau S stain). The lower image is using the same samples in a collagen type I zymogram.

b

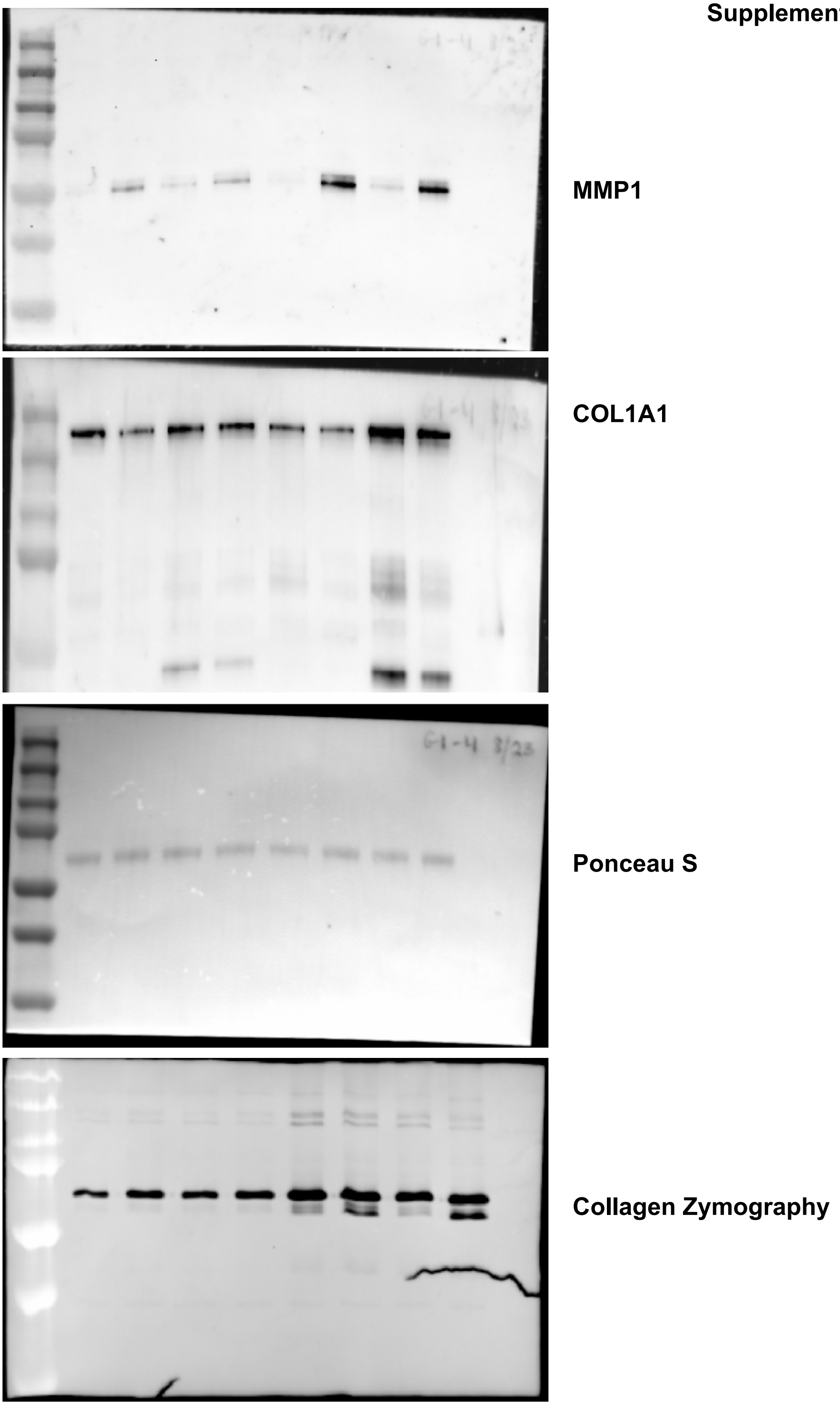

Supplemental Figure 15: Full length blots to accompany Figure 3B. Molecular weight marker is shown in the far left lane. The first 3 blot images are from the same membrane showing MMP1, Collagen 1A1 and total protein (Ponceau S stain). The lower image is using the same samples in a collagen type I zymogram.

Supplemental Figure 16

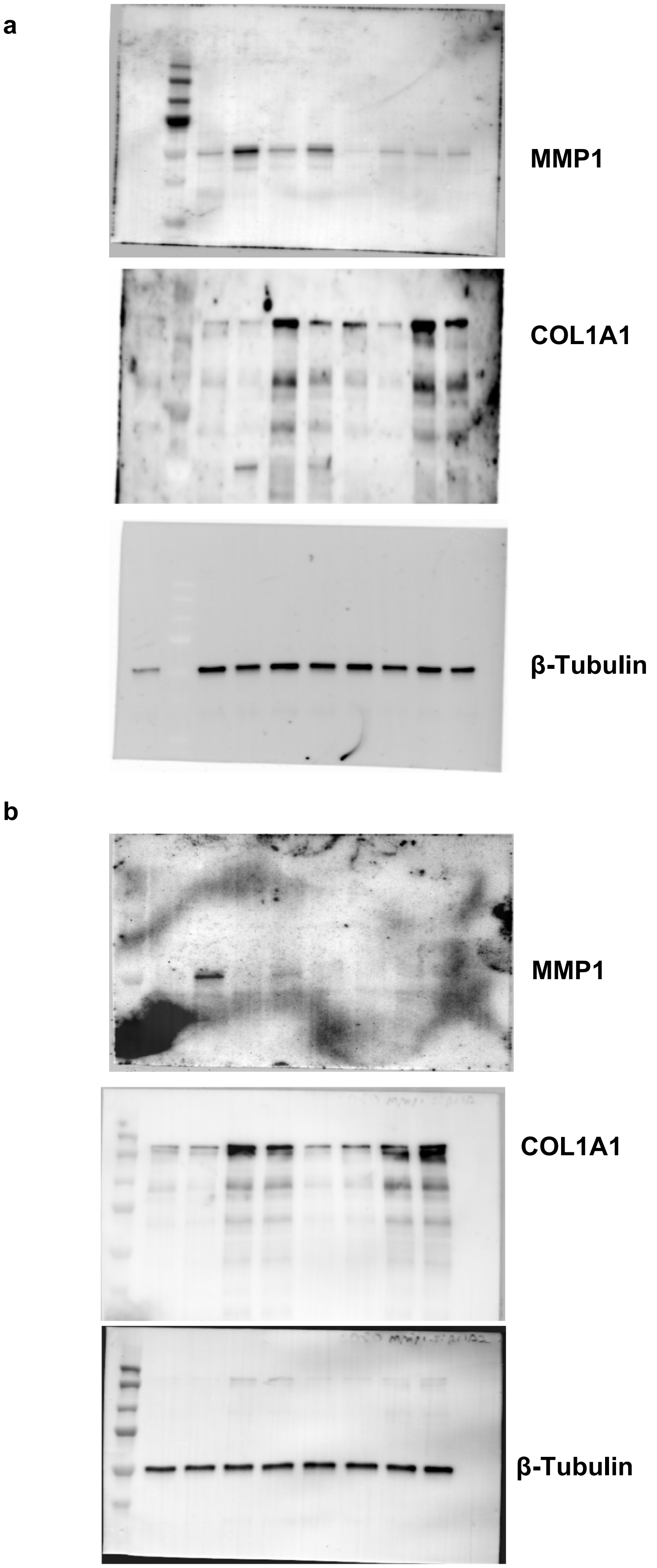

Supplemental Figure 16: Full length blots to accompany Figure 4. Full length blots corresponding to Figures 4a and 4b showing MMP1, Collagen 1A1 and β-tubulin in two different strains.

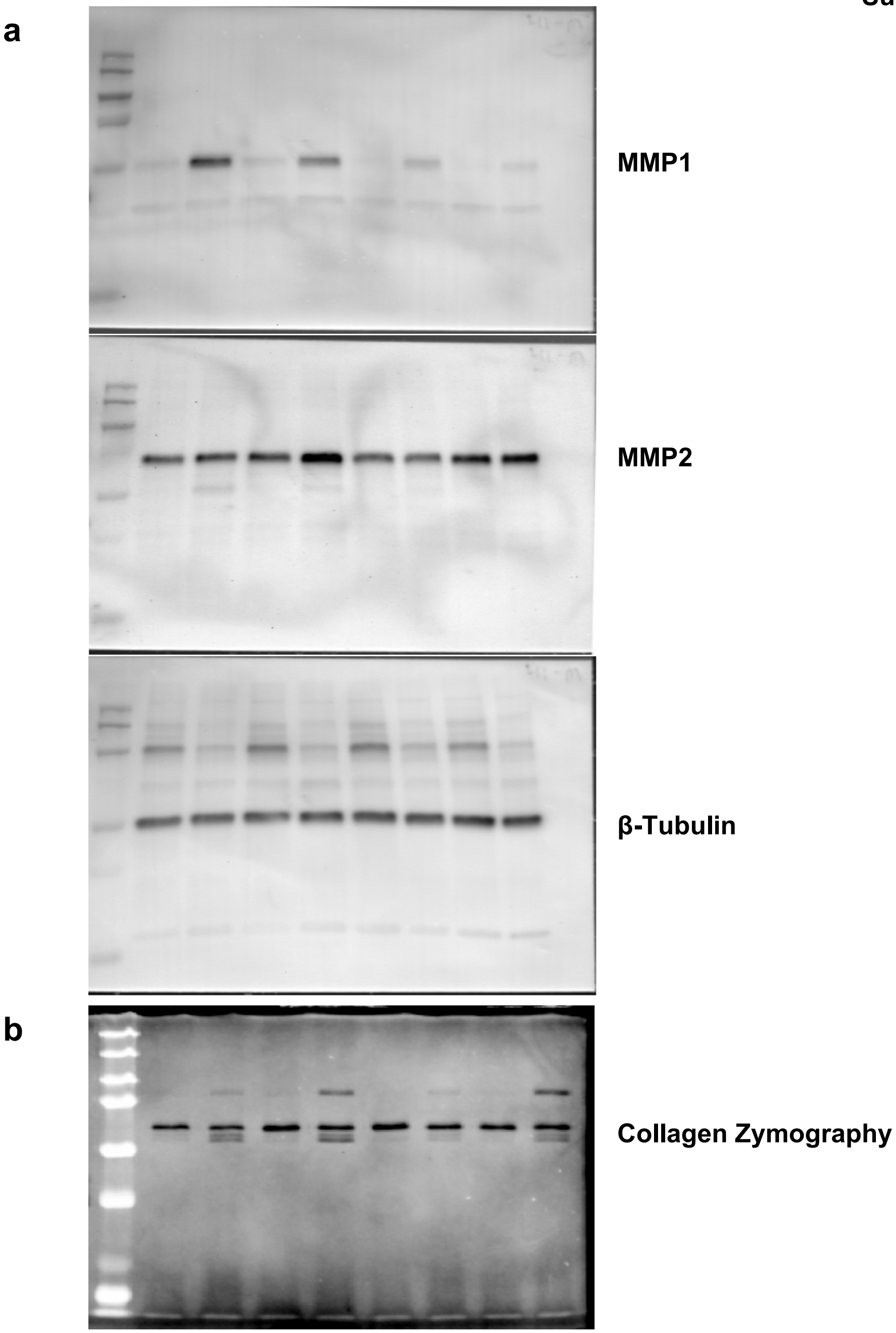

Supplemental Figure 17: Full length blots to accompany Figure 5. Molecular weight marker is shown in the far left lane. The first 3 blot images are from the same membrane showing MMP1, MMP2 and β-tubulin. The lower image issuing the same samples in a collagen type I zymogram.

Supplemental Figure 18

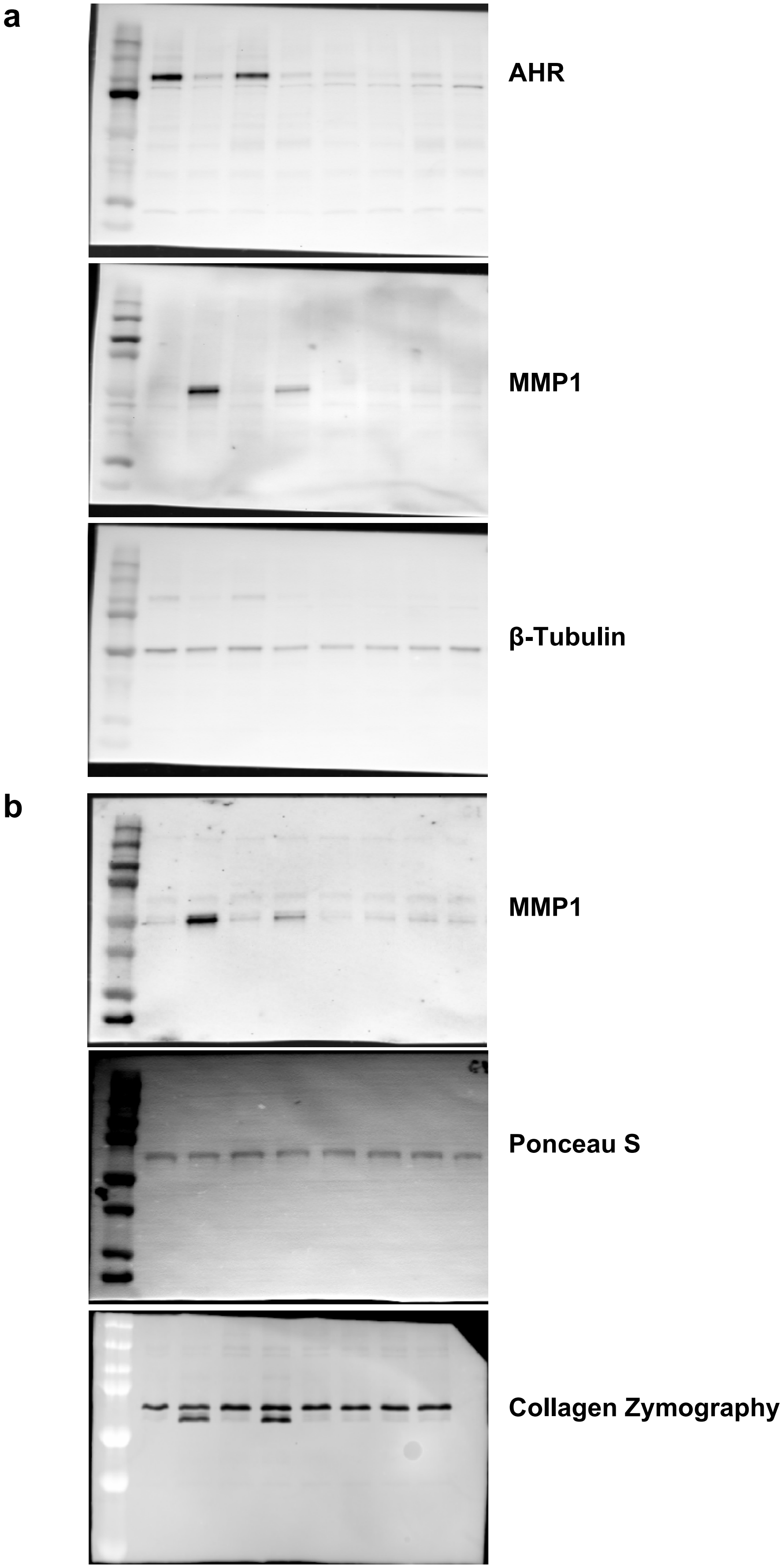

Supplemental Figure 18: Full length blots to accompany Figure 6. The same blot was used in **(a)** for AHR, MMP1, MMP2 and β-tubulin. The same samples were used in **(b)** for both Western and Zymography.

Supplemental Figure 19

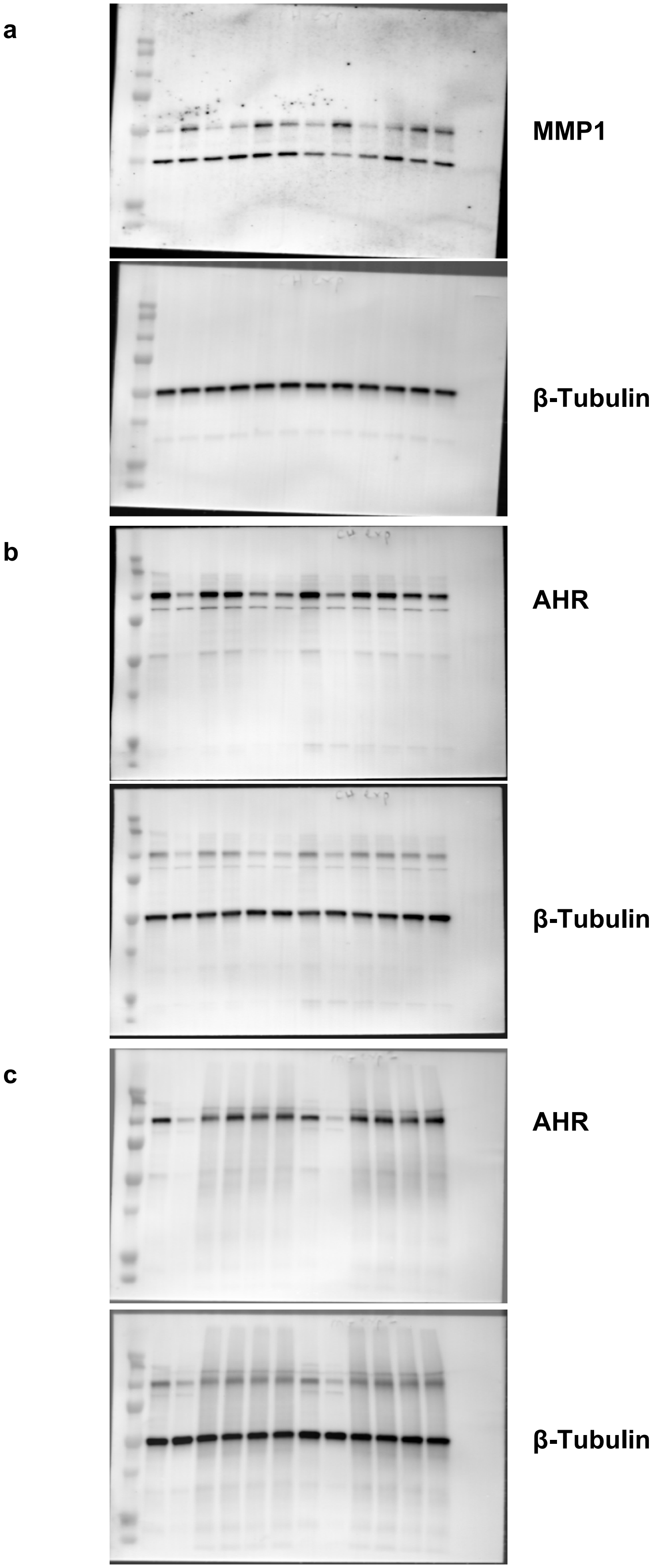

Supplemental Figure 19: Full length blots to accompany Figure 7. The same blot was used in **(a)** for AHR and β-tubulin. The same blot was used for MMP1 and β-tubulin **(b)**. In **(c)** the same blot was used for AHR and β-tubulin.

Supplemental Figure 20

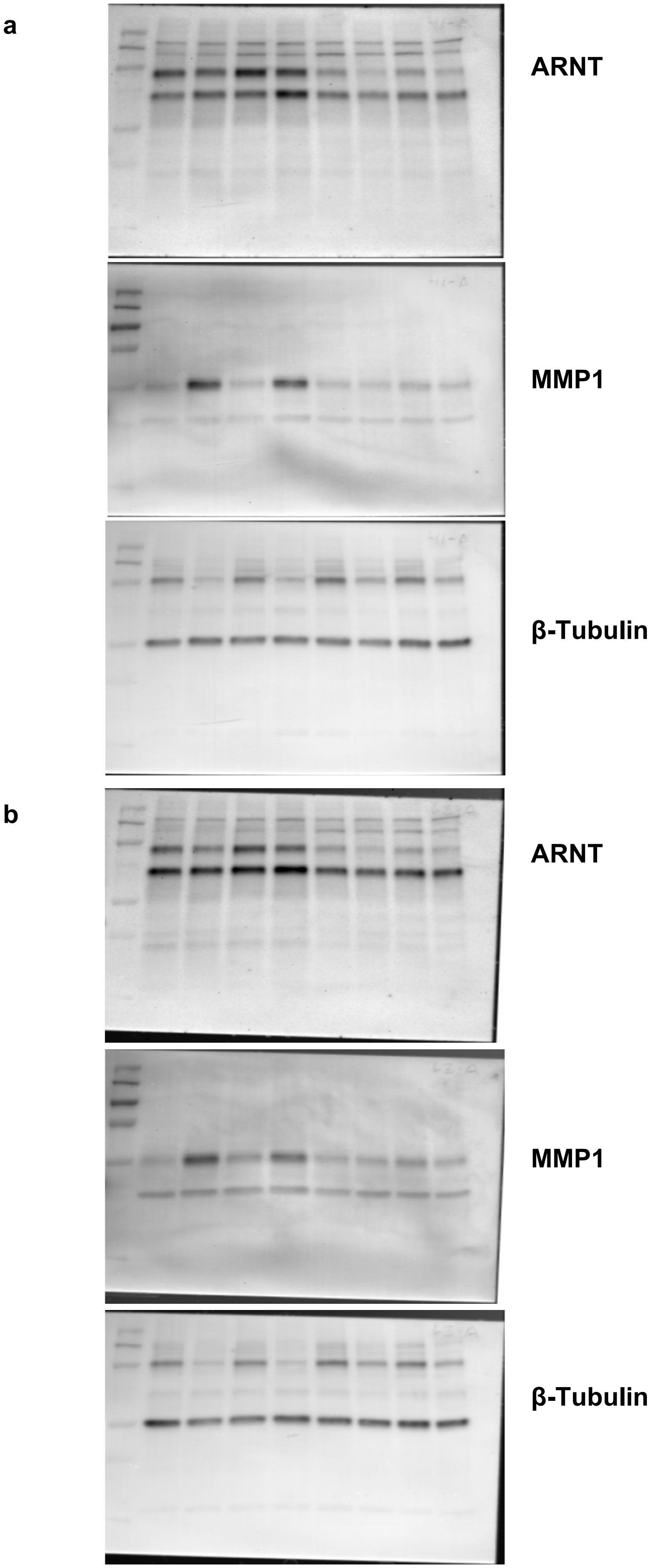

Supplemental Figure 20: Full length blots to accompany Figure 8. The same blot was used in **(a)** for ARNT, MMP1 and β-tubulin. Likewise, the same blot was used in **(b)** for ARNT, MMP1 and β-tubulin.

Supplemental Figure 21

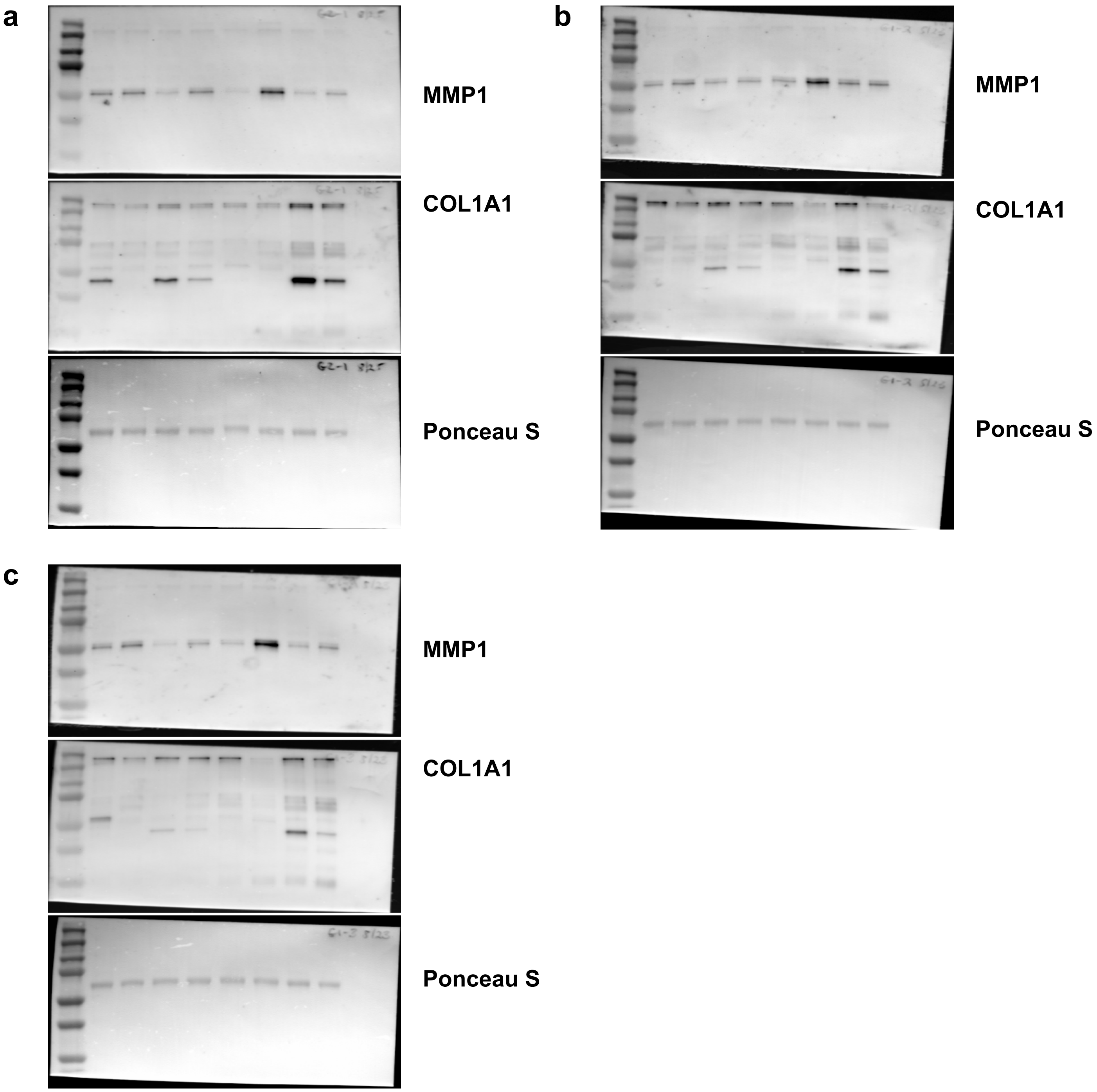

Supplemental Figure 21: Full length blots to accompany Supplemental Figure 1.

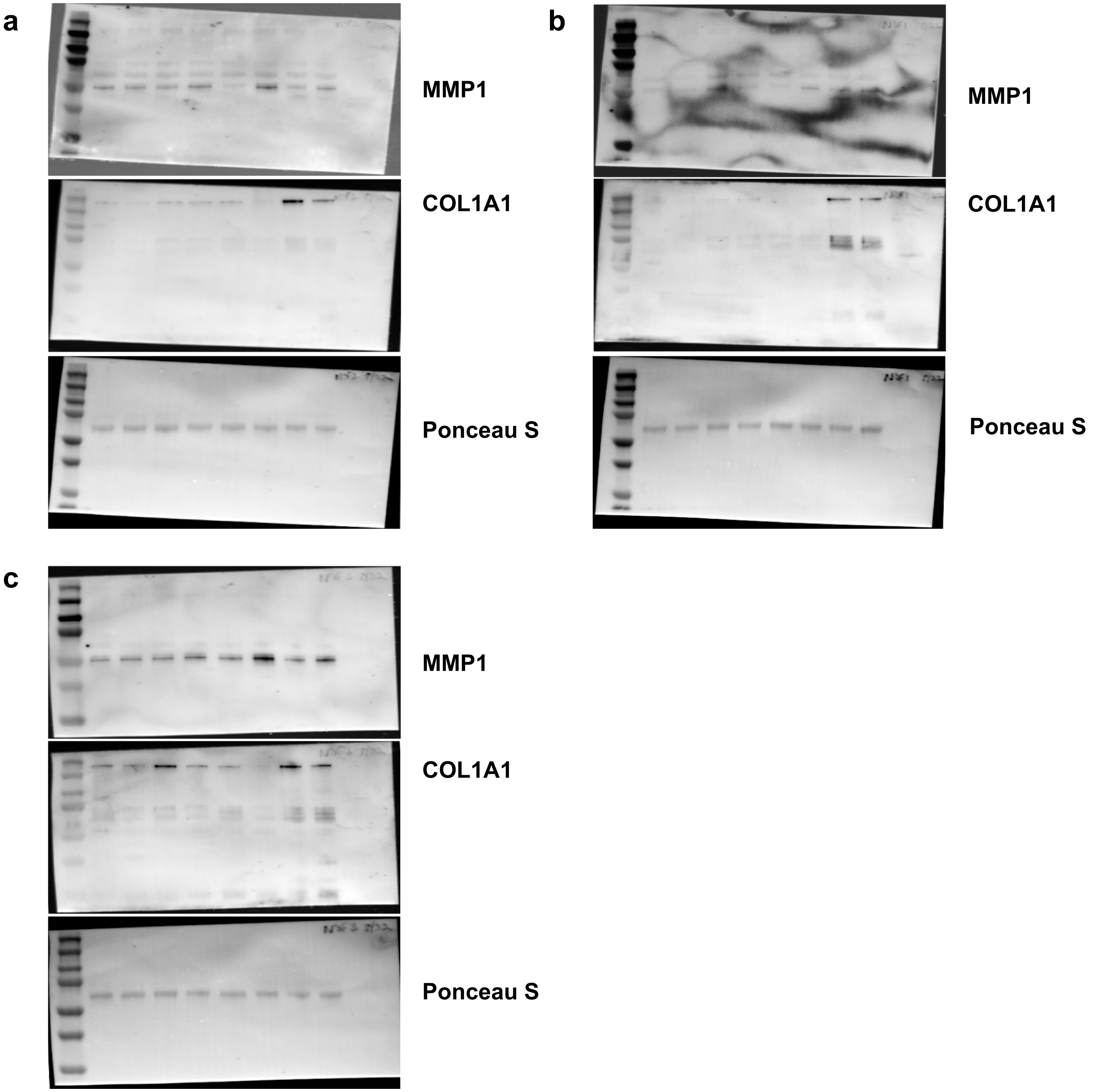

Supplemental Figure 22: Full length blots to accompany Supplemental Figure 2.

a

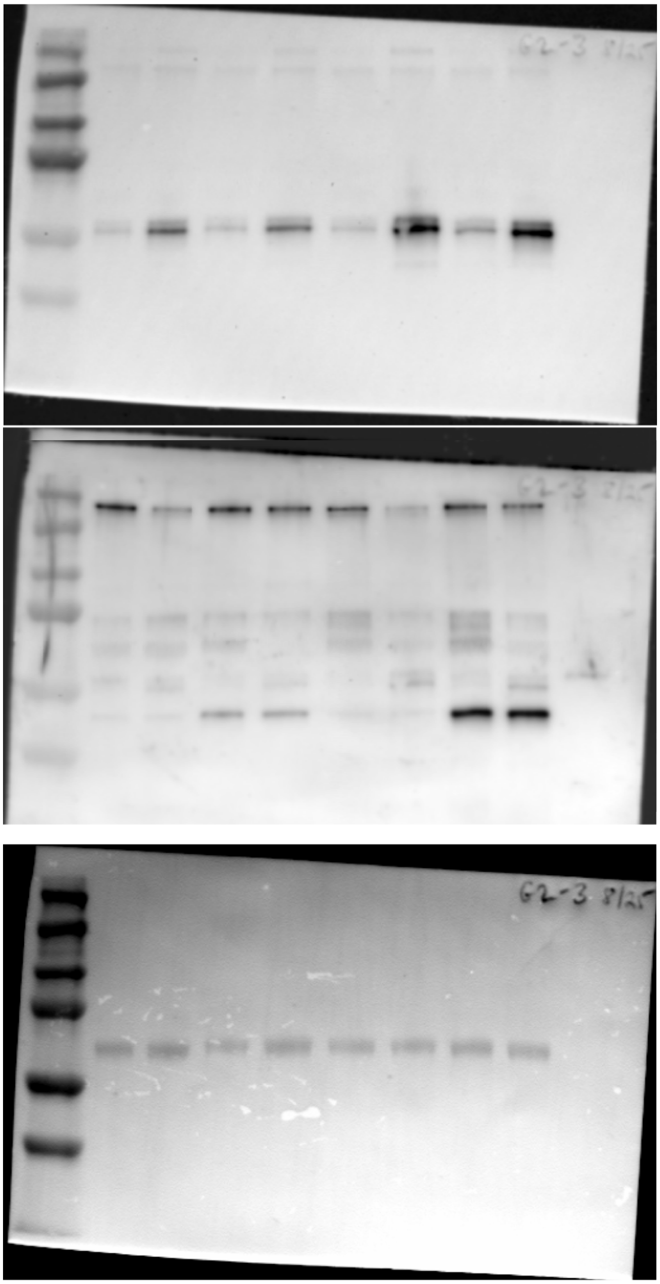

MMP1

COL1A1

Ponceau S

b

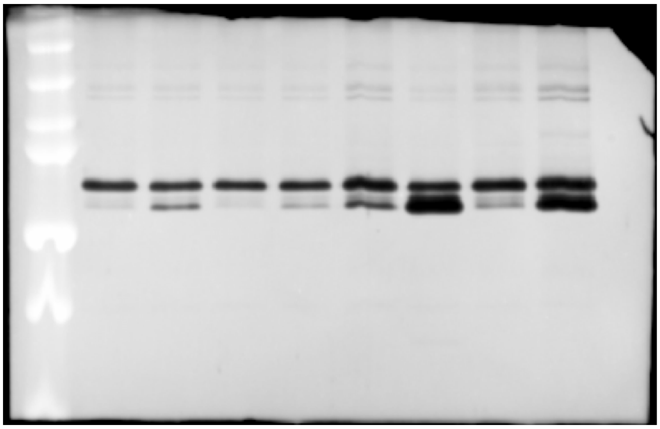

Collagen Zymography

Supplemental Figure 23: Full length blots to accompany Supplemental Figure 3.

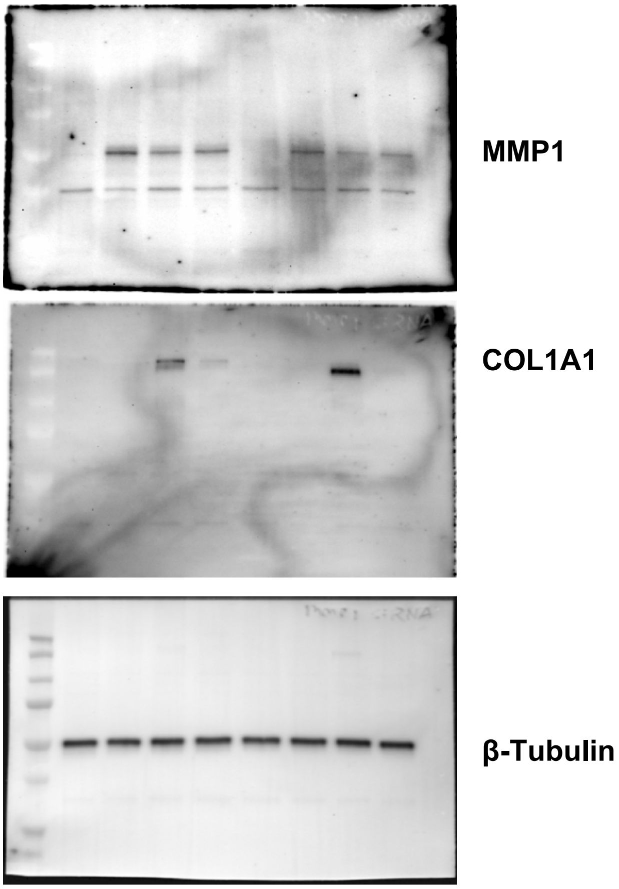

Supplemental Figure 24: Full length blots to accompany Supplemental Figure 4.

Supplemental Figure 25

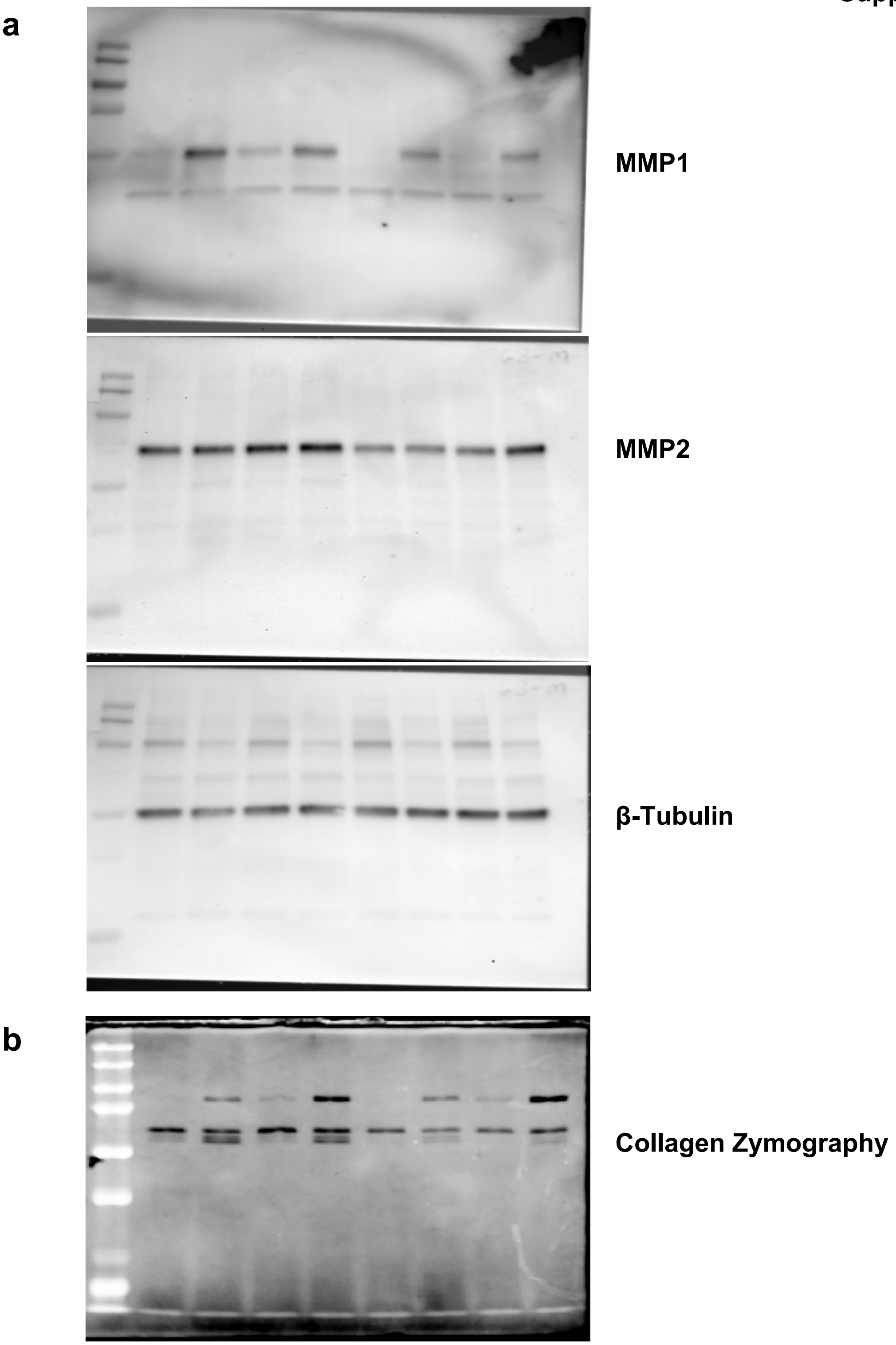

Supplemental Figure 25: Full length blots to accompany Supplemental Figure 5.

a

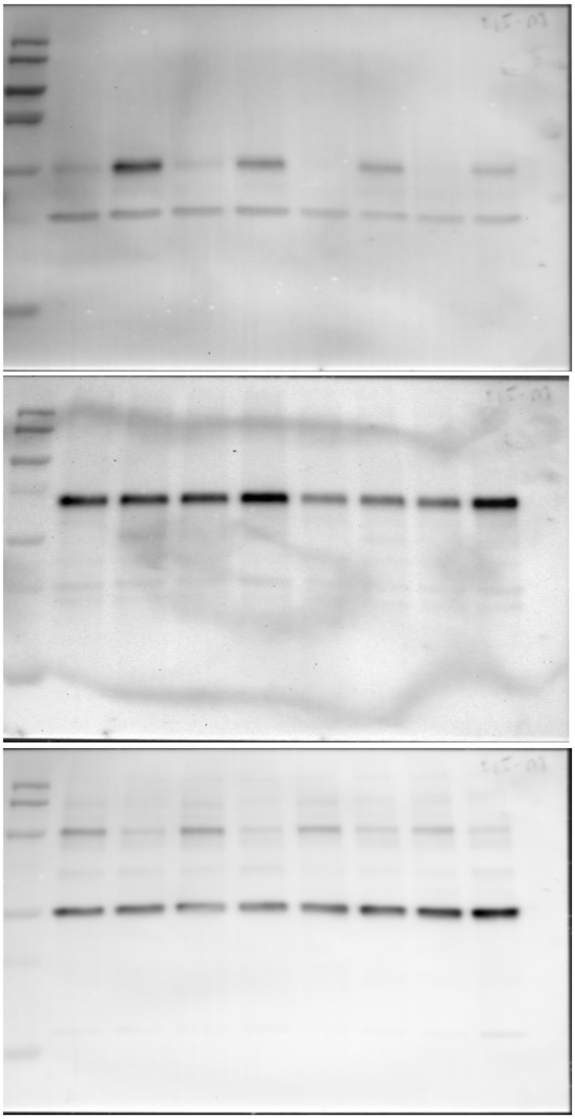

MMP1

MMP2

β-Tubulin

b

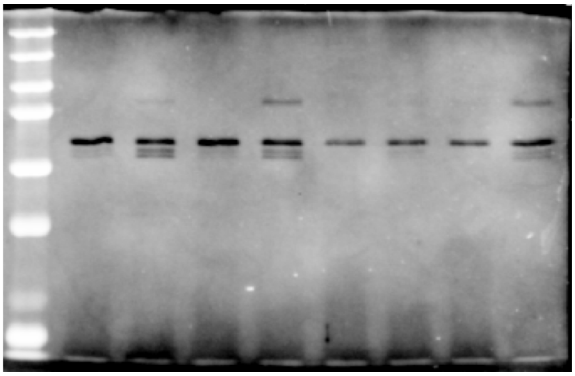

Collagen Zymography

Supplemental Figure 26: Full length blots to accompany Supplemental Figure 6.

Supplemental Figure 27

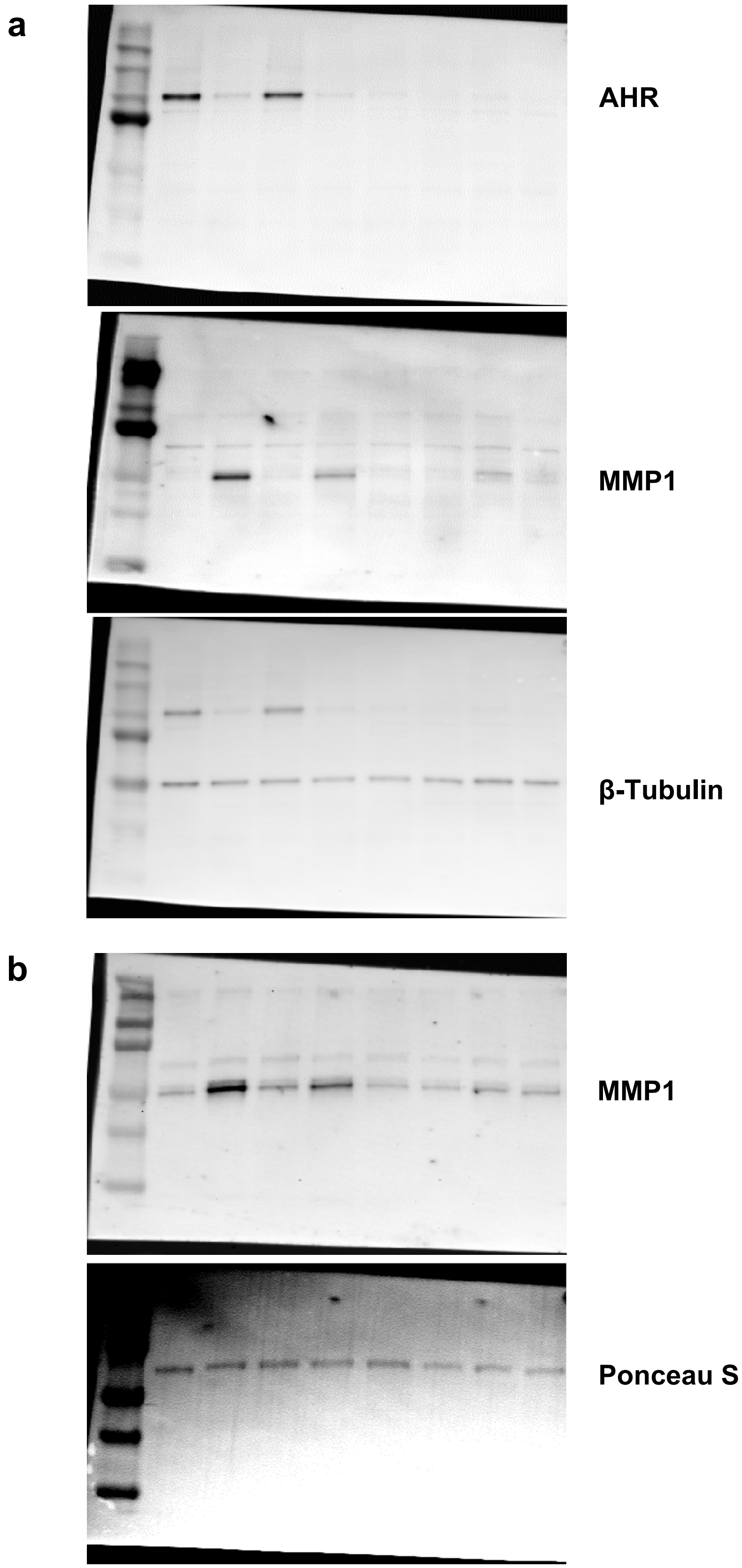

Supplemental Figure 27: Full length blots to accompany Supplemental Figure 8.

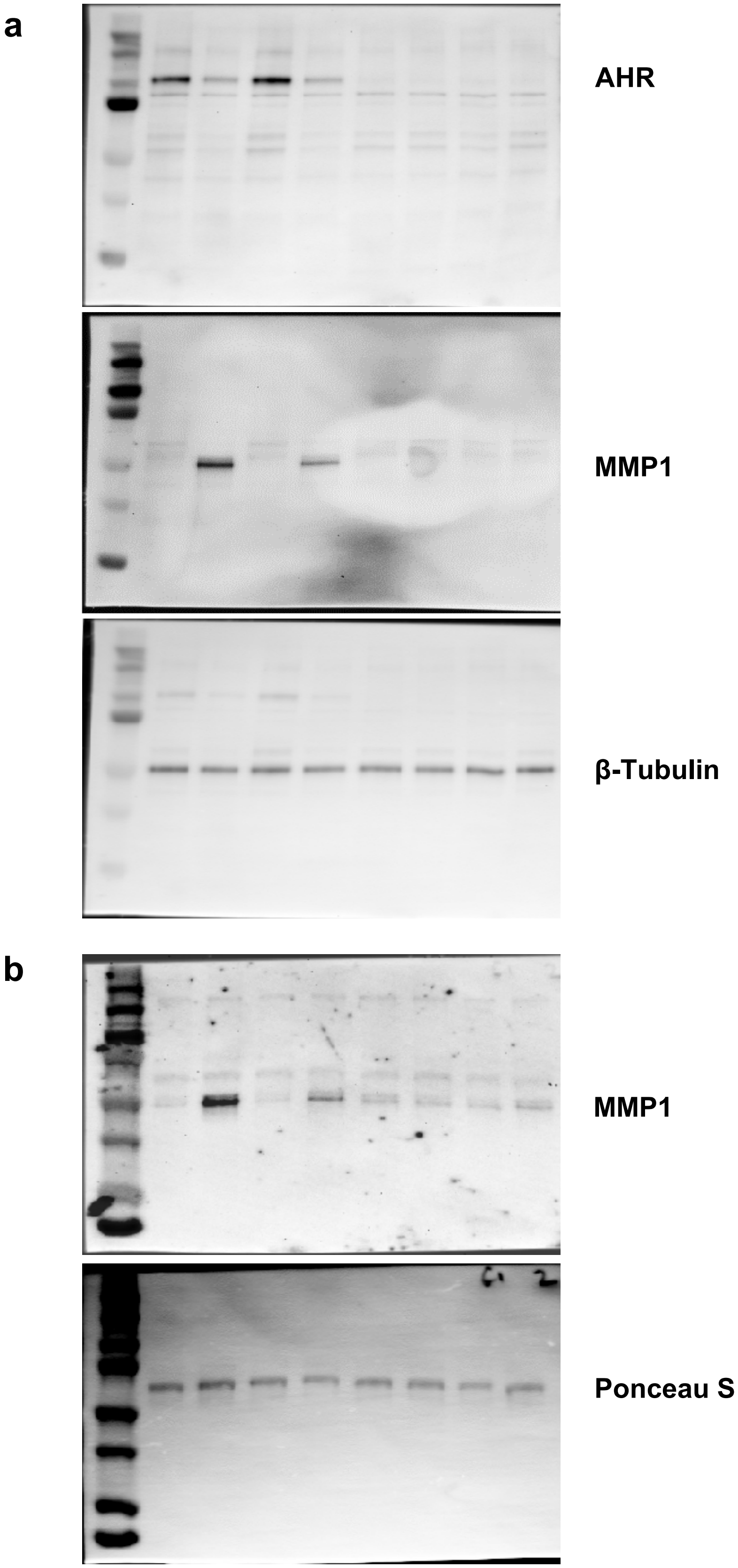

Supplemental Figure 28: Full length blots to accompany Supplemental Figure 9.

Supplemental Figure 29

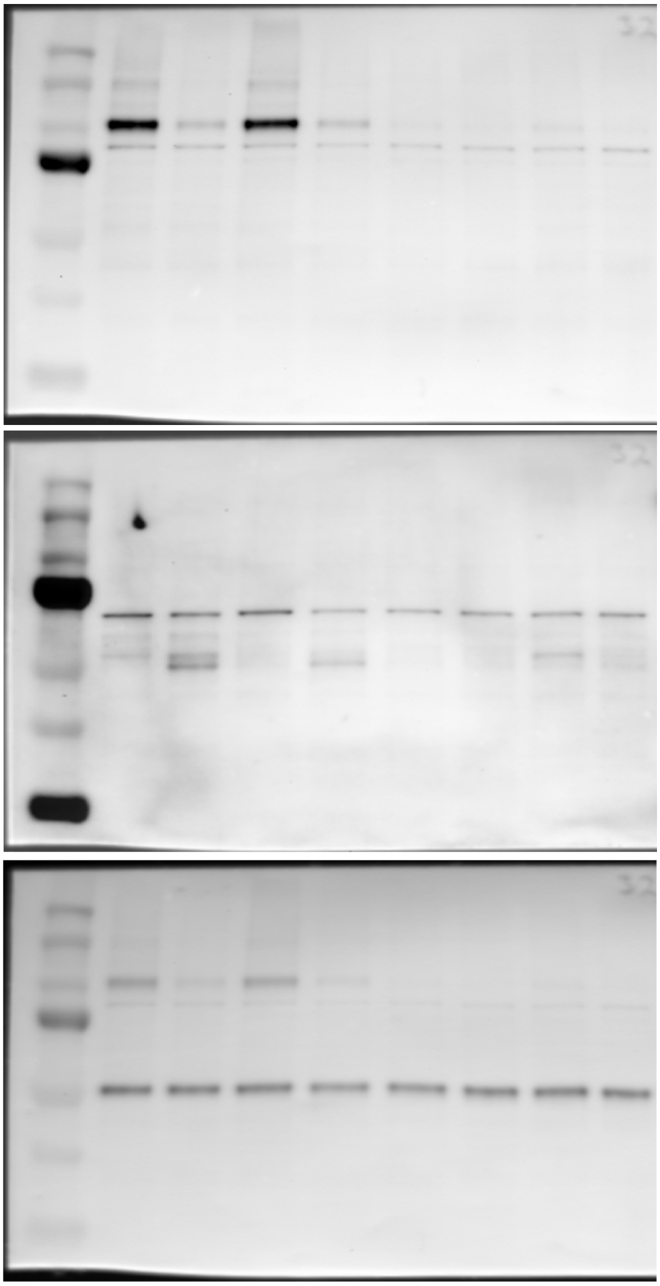

AHR

MMP1

β-Tubulin

Supplemental Figure 29: Full length blots to accompany Supplemental Figure 10.

a

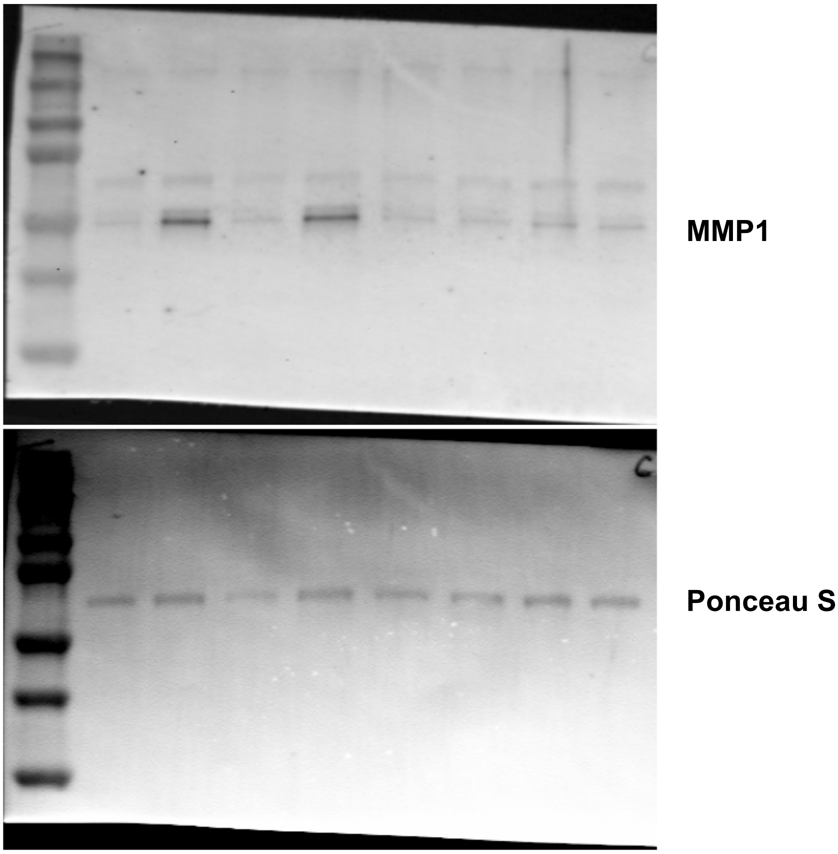

b

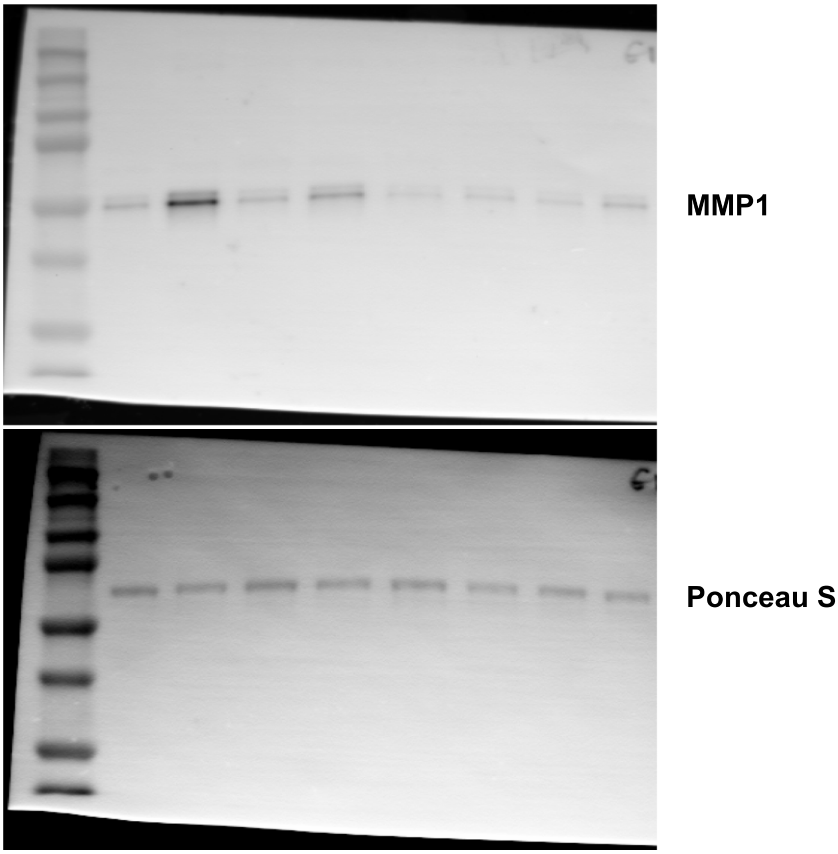

Supplemental Figure 30: Full length blots to accompany Supplemental Figure 11.

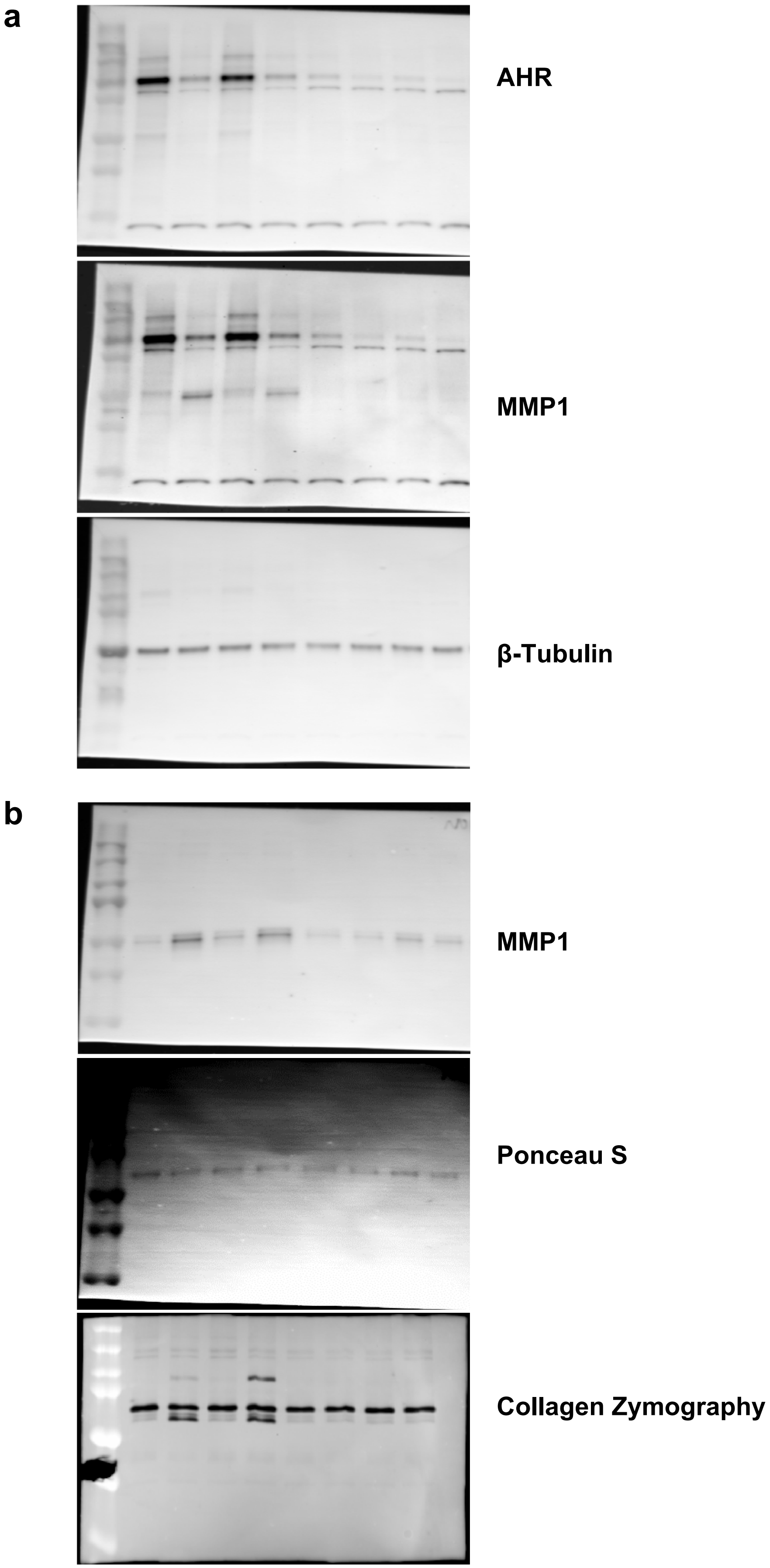

Supplemental Figure 31: Full length blots to accompany Supplemental Figure 12.

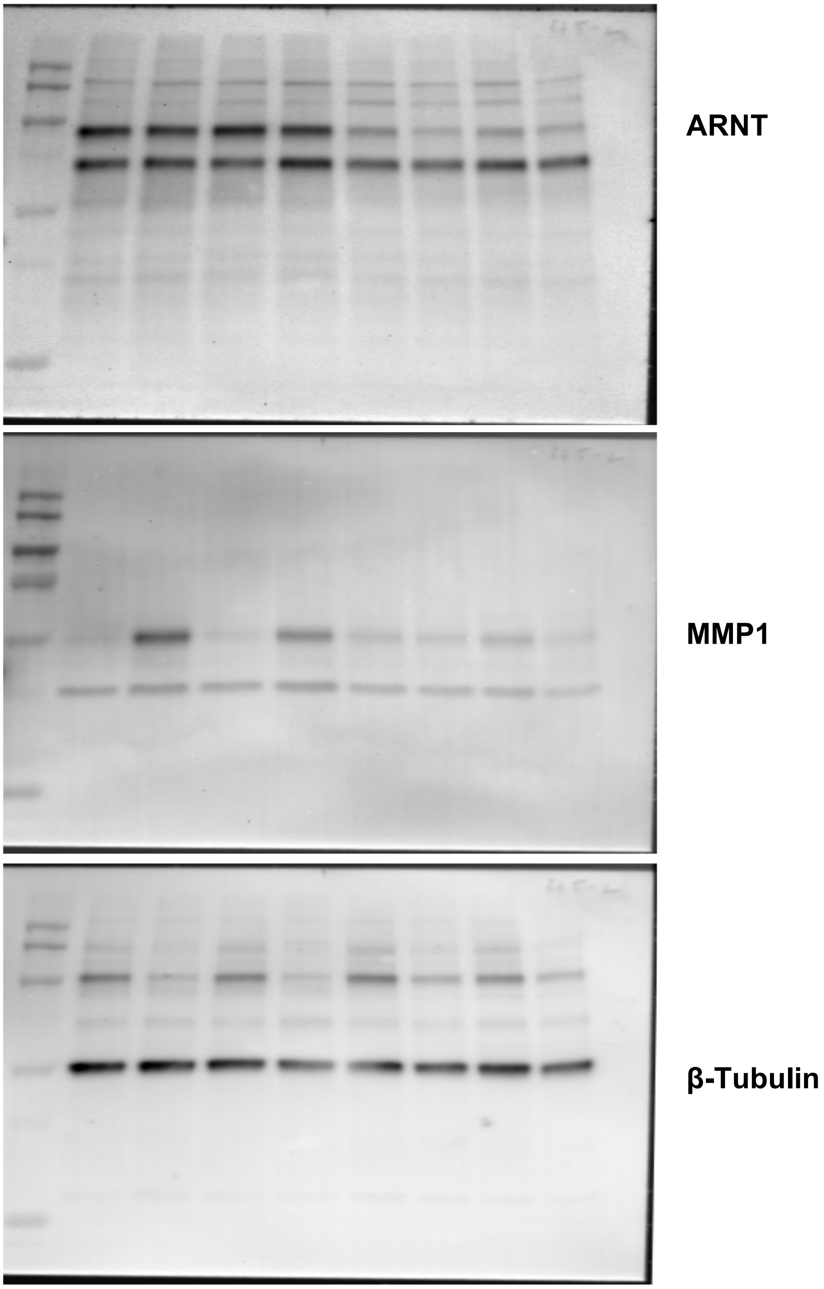

Supplemental Figure 32: Full length blots to accompany Supplemental Figure 13.
